# The Histidine Kinase CckA Is Directly Inhibited by a Response Regulator-like Protein in a Negative Feedback Loop

**DOI:** 10.1128/mbio.01481-22

**Published:** 2022-07-25

**Authors:** Benjamín Vega-Baray, Clelia Domenzain, Sebastián Poggio, Georges Dreyfus, Laura Camarena

**Affiliations:** a Instituto de Investigaciones Biomédicas, Universidad Nacional Autónoma de México, Mexico City, Mexico; b Institituto de Fisiología Celular, Universidad Nacional Autónoma de México, Mexico City, Mexico; National Cancer Institute

**Keywords:** Rhodobacter sphaeroides, CckA, two-component systems, Osp, bacterial signal transduction, hybrid histidine kinase, *Roseobacteraceae*

## Abstract

In alphaproteobacteria, the two-component system (TCS) formed by the hybrid histidine kinase CckA, the phosphotransfer protein ChpT, and the response regulator CtrA is widely distributed. In these microorganisms, this system controls diverse functions such as motility, DNA repair, and cell division. In *Caulobacterales* and *Rhizobiales*, CckA is regulated by the pseudo- histidine kinase DivL, and the response regulator DivK. However, this regulatory circuit differs for other bacterial groups. For instance, in *Rhodobacterales,* DivK is absent and DivL consists of only the regulatory PAS domain. In this study, we report that, in Rhodobacter sphaeroides, the kinase activity of CckA is inhibited by Osp, a single domain response regulator (SDRR) protein that directly interacts with the transmitter domain of CckA. *In vitro*, the kinase activity of CckA was severely inhibited with an equimolar amount of Osp, whereas the phosphatase activity of CckA was not affected. We also found that the expression of *osp* is activated by CtrA creating a negative feedback loop. However, under growth conditions known to activate the TCS, the increased expression of *osp* does not parallel Osp accumulation, indicating a complex regulation. Phylogenetic analysis of selected species of *Rhodobacterales* revealed that Osp is widely distributed in several genera. For most of these species, we found a sequence highly similar to the CtrA-binding site in the control region of *osp*, suggesting that the TCS CckA/ChpT/CtrA is controlled by a novel regulatory circuit that includes Osp in these bacteria.

## INTRODUCTION

In bacteria, two component systems (TCS) are used to perceive and transduce many different input signals and provide adaptive responses to extracellular and intracellular cues. In its simplest form, TCS are formed by a sensor histidine kinase (SHK) and a response regulator (RR). The stimulus perceived by a specific sensor domain of the SHK, results in autophosphorylation of the conserved histidine (H) residue present in the transmitter domain, which includes both the catalytic (CA), and the dimerization and histidine phosphotransfer domains (DHp). The phosphoryl group is transferred to a conserved aspartic acid (D) residue present in the receiver domain (REC) of the RR, which elicits an appropriate cellular response. Frequently the RR protein is a transcription factor that modifies the expression of a set of genes to accomplish the proper response. A variation of this basic scheme involves hybrid histidine kinases (SHHK) in which a REC domain is fused to the SHK; in these cases, the presence of an additional phosphotransfer (HPt) domain that either can be an independent polypeptide or be part of the SHHK, is required to achieve phosphorylation of the RR protein (1–3).

The TCS formed by the membrane SHHK CckA, the Hpt protein ChpT, and the RR CtrA is widely distributed in alphaproteobacteria (4), and it has been extensively characterized in the dimorphic bacterium Caulobacter crescentus where progression of its cell cycle is controlled by CtrA (5–7). In this bacterium, each cell division is asymmetrical, resulting in a swarmer cell unable to replicate its DNA and a replicatively active stalked cell. After division, the stalked cell can initiate a new cycle of DNA replication, while the swarmer cell needs to differentiate into a stalked cell after a determined period of time. Cell cycle progression is controlled by a complex program in which the CckA/ChpT/CtrA system integrates the information from different regulatory proteins (8). The temporal and spatial presence of the phosphorylated form of CtrA (CtrA-P) controls the fate of the daughter cells by activating and repressing genes with critical roles in cell cycle progression and cell development (9, 10).

In C. crescentus, different proteins control the output of this TCS. These regulators alter CtrA stability, determine if CckA functions as a kinase or a phosphatase and, in consequence, control the spatial distribution of CtrA-P (11–15). One of these regulatory modules consists of the pseudo-HK DivL, the RR DivK, and the kinase/phosphatase DivJ and PleC proteins (16–18). Specifically, DivL stimulates the kinase activity of CckA in the flagellated pole, where the allosteric regulator of DivL, named DivK, is actively dephosphorylated by PleC. In contrast, in the stalked cell pole, DivK is maintained in its phosphorylated form by DivJ (19). The interaction between DivK-P and DivL alters the CckA-DivL interaction and favors the phosphatase activity of CckA (16, 17, 20). In addition, the second messenger c-di-GMP that drives the swarmer-stalked transition binds directly to CckA switching its activity from kinase to phosphatase (21, 22).

A bioinformatic analysis revealed that several regulators of CckA such as DivJ and DivK, are absent in *Rhodobacterales*, *Rickettsiales,* and several species of *Rhodospirillales*, suggesting that CckA could be controlled by other proteins (4).

In Rhodobacter sphaeroides the TCS CckA/ChpT/CtrA is required for the expression of the Fla2 flagellar system (23). This bacterium has two different flagellar systems of different phylogenetic origin, which are controlled by different transcription factors (23–26). Under the standard growth conditions used in the laboratory, only the single subpolar Fla1 flagellum is assembled whereas the *fla2* genes are not expressed, indicating that the TCS CckA/ChpT/CtrA is inactive (24). Expression of the *fla2* genes has been reported in double mutants that carry a gain of function mutation in CckA, and another mutation that blocks the synthesis of the Fla1 flagellum. A single mutation preventing the expression of the *fla1* genes does not result in the expression of *fla2* (23, 24, 27, 28).

Transcriptomic profiling of the genes controlled by CtrA in R. sphaeroides revealed that at least 321 genes are regulated by CtrA, which are distributed across many functional categories. In particular, CtrA affects specific pathways such as *fla2*-dependent motility, chemotaxis, gas vesicle formation, photosynthesis, etc. (29). In contrast to many studied species of α- proteobacteria, in R. sphaeroides, this TCS is not essential and, in fact, its expression is turned off under many different growth conditions. The signals that activate or repress CckA/ChpT/CtrA in this bacterium are largely unknown, but it has been reported that photoheterotrophic growth using a poor carbon source in the culture medium such as 0.1 mM succinic acid or cas amino acids favors activation of the system (23, 30).

In this study, we report the existence of a new type of CckA regulator that directly inhibits its kinase activity by binding to its transmitter domain. In its absence, activation of CckA brings about the expression of the genes activated by CtrA-P. The wide distribution of the gene encoding this negative regulator across *Rhodobacterales* suggests that this mechanism of regulation is prevalent in several genera of this Order. A comprehensive characterization of the role of this protein in R. sphaeroides is presented in this study.

## RESULTS

### Isolation of mutant strains with an active CckA/ChpT/CtrA TCS.

**FIG 1:**
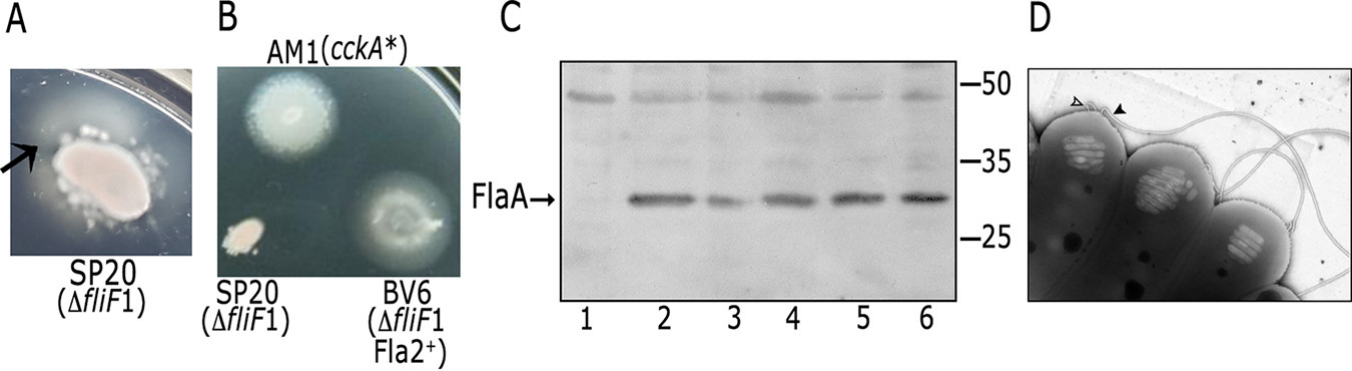
(A) Isolation of a spontaneous Fla2 mutant + from a non-motile strain (SP20) inoculated on a soft agar plate and incubated for 7 days at 30°C. The arrow indicates the bulge caused by the swimmer cells emerging from the colony. (B) Swimming phenotype of the Fla2+ mutant strain BV6 after purification, as controls strains AM1 and SP20 were included. The AM1 strain expresses the constitutive version of CckA, CckA_L391F_. The gene encoding this mutant version of CckA is represented as cckA*. Plates containing Sistrom’s minimal medium with 0.1 mM succinic acid as a carbon source were inoculated with cells from a saturated culture and incubated for 60 h. (C) Anti-FlaA Western blot analysis of total cell extracts of strains LC7 (Δ*ctrA*::Hyg) (lane 1), AM1 (lane 2), BV6 to BV9 (lanes 3 to 6). Migration of the molecular mass markers is shown at the right and values expressed in kDa. (D) Transmission electron microscopy of BV6 cells showing the presence of flagella. For the cell on the left, the flagellar filament is indicated with a black arrowhead and an open arrowhead indicates the presence of two flagellar hooks that remain attached to the cell body when the flagellar filament was broken during manipulation.

To obtain new insights regarding the mechanisms that control activation of the CckA/ChpT/CtrA TCS in R. sphaeroides, we isolated mutants that had an altered output of the system. We took advantage that under the growth conditions commonly used in the laboratory, this TCS is turned off, so we proceeded to select mutant strains able to swim with the Fla2 flagellum. For this, a mutant strain in the master regulator of the Fla1 system, FleQ (SP13 strain) (25), or a mutant defective in an early protein required for Fla1 biogenesis, such as the membrane protein FliF (SP20 strain, *fliF*1::*aadA*), were inoculated on soft agar plates. These strains are non-motile; however, after 7 days of incubation, irregular flares emerged, indicating the presence of motile cells (Fig. 1A). Four independent isolates, two from each parental strain, were selected and purified; these strains were inoculated on soft agar plates, and it was observed that they spread uniformly, indicating the presence of a homogeneous population (Fig. 1B). For comparison, the AM1 strain carrying a gain of function version of CckA (CckA_L391F_, and labeled in Fig. 1B as cckA*) was used as a positive control (23, 27). Western blotting of total cell extracts of these strains revealed the presence of the Fla2 flagellar filament protein (FlaA) (Fig. 1C). Therefore, the phenotype of these spontaneous mutants was assigned as Fla2+. Electron microscopy analysis of one of these Fla2+ strains, BV6, revealed the presence of polar flagella, a distinctive feature of the Fla2 flagellation pattern (Fig. 1D). For AM1 and other strains with an active Fla2 system, it has been reported the presence of several polar flagella with an average of 4.5 flagella per cell (24, 31). In Fig. 1D, it was also possible to observe the presence of gas vesicles, detected as electron-lucent bodies in the cytoplasm. It was previously shown that the formation of these structures is also dependent on CtrA (29).

Formation of Fla2 flagella indicates that the TCS CckA/ChpT/CtrA is active in the BV6 strain and strongly suggests that, in the other strains that were isolated, this would also be the case. However, no mutations were found after sequencing *cckA*, *chpT* and *ctrA*, in BV6 to BV9 strains. Therefore, the complete genome sequence of the BV6 strain was obtained and compared with the genome sequence of the wild-type WS8N strain. From this analysis, the only mutation identified corresponds to a transversion in the gene RSWS8N_09785 that encodes for a protein of 120 residues that is predicted to be a SDRR. This mutation causes the substitution of His115 for Asp.

### The absence of RSWS8N_09785 is responsible of the activation of the TCS CckA/ChpT/CtrA.

We learned that the RSWS8N_09785 homologous gene in R. sphaeroides 2.4.1 was previously reported to be a positive regulator of photosynthesis but in that report, no relationship with the TCS CckA/ChpT/CtrA was established. This gene was named *osp* that stands for optimal synthesis of the photosynthetic apparatus (32).

**FIG 2:**
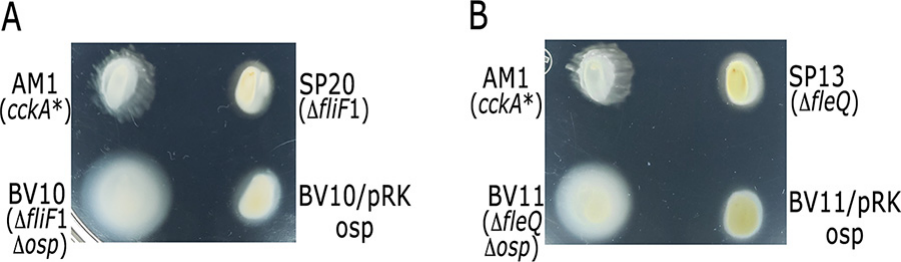
Swimming plates of mutant strains SP20 (A) and SP13 (B) carrying the *osp*::Hyg allele. Mutant strains were complemented using plasmid pRK_osp. Control strains carry the empty plasmid pRK415. The AM1 strain expresses the constitutive version of CckA, CckA_L391F_. The gene encoding this mutant version of CckA is represented as cckA*. Plates containing Sistrom’s minimal medium supplemented with 1 μg mL^−1^ tetracycline and 0.1 mM succinic acid as a carbon source were incubated for 60 h under photoheterotrophic conditions. The diameter of the swimming rings was determined from at least three independent experiments. For panel A, AM1 = 1.85 cm SD ± 0.11; SP20 = 1.3 cm SD ± 0.04; BV10 = 2.58 cm SD ± 0.12; BV10/pRK_osp = 1.48 cm SD ± 0.05. A significant difference of *P < *0.01 for SP20, BV10 and BV10/pRK_osp versus AM1 and BV10 versus BV10/pRK_osp was determined by one-way analysis of variance. For panel B, AM1 = 1.87 cm SD ± 0.03; SP13 = 1.33 cm SD ± 0.14; BV11 = 1.97 cm SD ± 0.05; BV11/pRK_osp = 1.49 cm SD ± 0.19. A significant difference of *P < *0.01 for SP13, and BV11/pRK_osp versus AM1; and BV11 versus BV11/pRK_09785 was determined using the same statistical test.

Therefore, to ascertain that the product of RSWS8N_09785 from here on *osp* was related with the observed phenotypes, we replaced the chromosomal gene by the mutant allele *osp*::Hyg in the strain SP20. In contrast to the parental strain that was unable to swim, it was observed that the loss of *osp* makes swimming of the resultant strain possible (Fig. 2A). The introduction of the wild-type gene in plasmid pRK415 (pRK_osp) restores the parental phenotype confirming that the Osp protein is solely responsible of the observed phenotype in the original mutant strain (Fig. 2A). The same results were observed using SP13 as parental strain (Fig. 2B). It should be noted that the swimming ability of the strain carrying a mutation in *osp* was dependent on the presence of CckA, ChpT, and CtrA indicating that, in this strain, the Fla2+ phenotype is still dependent on the 3 components of the system (data not shown).

**FIG 3:**
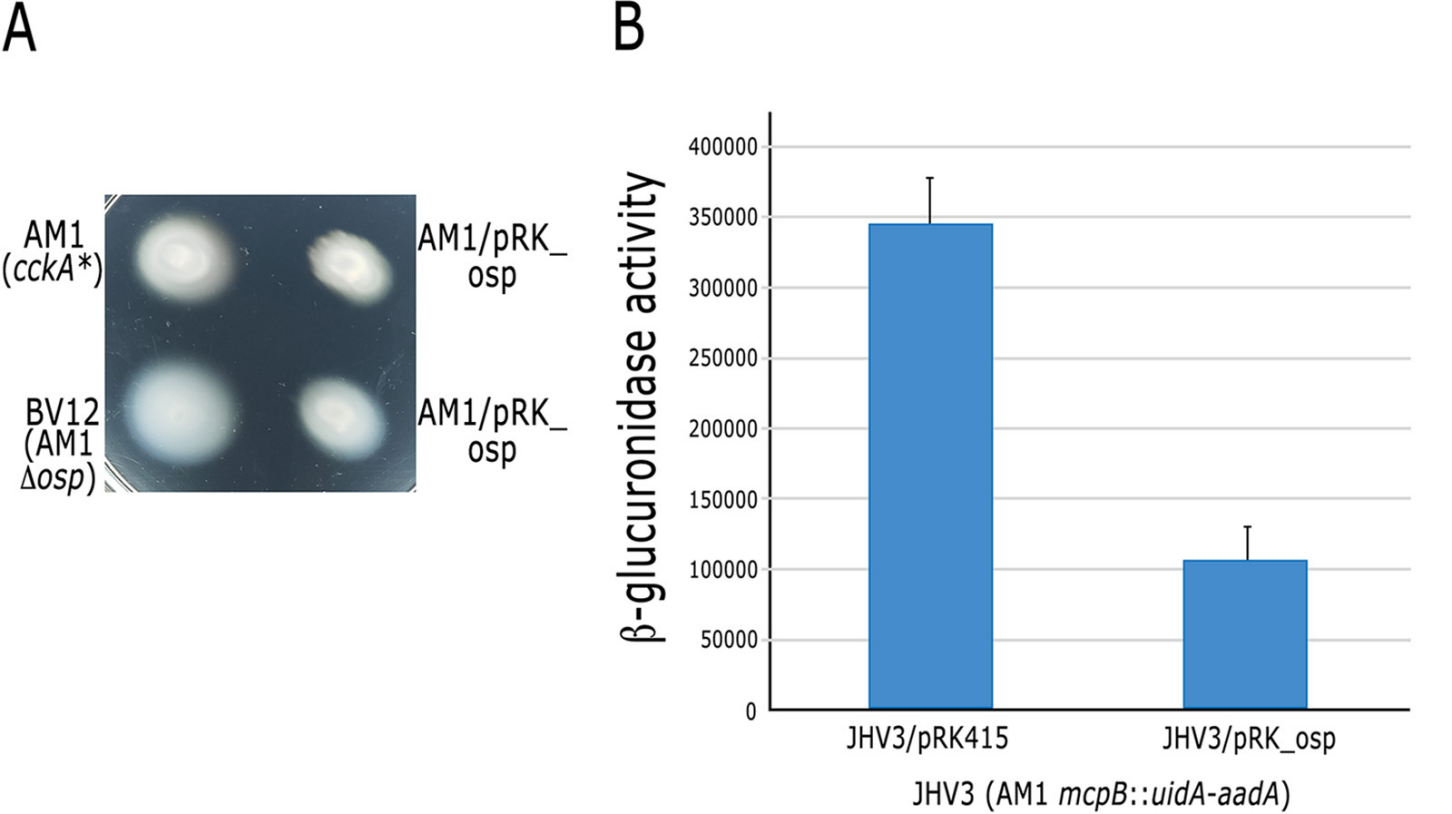
(A) Swimming plate of AM1 and its derivative BV12 (AM1 *osp*::Hyg) strains carrying an empty plasmid pRK415 or pRK_osp. Plates containing Sistrom’s minimal medium supplemented with 1 μg mL^−1^ tetracycline and 0.1 mM succinic acid as a carbon source were incubated for 60 h. The diameter of the swimming rings was determined from at least three independent experiments. AM1 = 1.89 cm SD ± 0.06; AM1/pRK_osp = 1.17 cm SD ± 0.05; BV12 = 2.59 cm SD ± 0.28; BV12/pRK_osp = 1.68 cm SD ± 0.24. A significant difference of *P < *0.01 for AM1/pRK_osp versus AM1; BV12/pRK_osp versus BV12 and BV12 versus AM1 was determined by one-way analysis of variance. (B) β-glucuronidase activity driven by the chromosomal fusion *mcpB*::*uidA*-*aadA* present in JHV3 was determined from strains carrying pRK415 or pRK_osp. Total cell extracts were obtained from cultures grown photoheterotrophically in Sistrom’s minimal medium supplemented with 0.2% cas amino acids as a carbon source. Activity is expressed as picomoles of methylumbelliferone formed per minute per milligram of protein. A significant difference of *P < *0.01 for JHV3/pRK415 versus JHV3/pRK_osp was determined using a two-tailed *t* test.

We also established that Osp negatively affects the gain of function version of CckA that is expressed in the AM1 strain (CckA_L391F_) given that swimming of these cells was severely reduced by the presence of a plasmid expressing this protein (Fig. 3A). Deletion of *osp* in AM1 cells caused a slight increment in swimming, and this effect was counteracted by the presence of pRK_osp (Fig. 3A).

The hypothesis that Osp is a negative regulator of the TCS CckA/ChpT/CtrA was additionally supported by measuring the expression of the CtrA-dependent *mcpB* gene in the AM1 derivative strain carrying the reporter fusion *mcpB*::*uidA*-*aadA* (29). *mcpB* is part of the chemotactic operon 1 (*che*Op1) and it was previously demonstrated that its expression is directly controlled by CtrA; therefore, it represents a reliable reporter of CtrA activation (26). As shown in Fig. 3B, β-glucuronidase activity (encoded by *uidA*) was severely reduced by the presence of pRK_osp. This result agrees with the notion that Osp limits CtrA activation.

In accordance with the idea that inactivation of *osp* induces the Fla2+ phenotype, we found that the remaining mutants also carried mutations in this gene. We observed for strain BV7 an insertion of a single nucleotide that shifted the open reading frame of *osp* generating a truncated protein of only 77 amino acids; for strain BV8 an insertion of 6 nucleotides that adds the amino acids A and V after residue 63, and for strain BV9 a deletion of a single nucleotide that shifted the open reading frame after residue 11. These strains were successfully complemented with the plasmid pRK_osp (data not shown).

### Osp is similar to a SDRR and its expression is dependent on CtrA.

**FIG 4:**
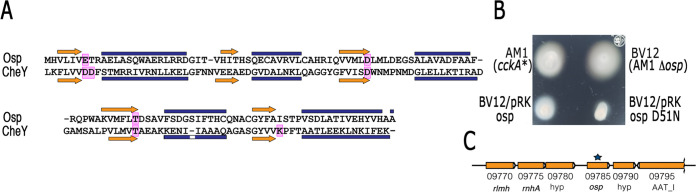
(A) Amino acid alignment of Osp and CheY from E. coli. The secondary structure features that conform the canonical structure of typical RRs is shown above and below the amino acid sequences of Osp and CheY. Conserved functional residues present in RRs are boxed in pink. The blue bars represent α-helixes, and yellow arrows β-strands. Secondary structure predictions were obtained using Psipred (84) and protein homology was evaluated using Swiss-Model (85) and the crystal structure of CheY (PDB 6TG7). (B) Swimming plate of BV12 strain carrying pRK_osp or pRK_osp D51N. Strains AM1 and BV12 carrying pRK415 were included as controls. Plates containing Sistrom’s minimal medium supplemented with 1 μg mL^−1^ tetracycline and 0.1 mM succinic acid as a carbon source were incubated for 60 h. The diameter of the swimming rings was determined from at least three independent experiments, AM1 = 1.82 cm SD ± 0.13; BV12 = 2.5 cm SD ± 0.25; BV12/pRK_osp = 1.64 cm SD ± 0.25; BV12/pRK_osp D51N = 1.12 cm SD ± 0.1. A significant difference of *P < *0.01 for BV12/pRK_osp and BV12/pRK_osp D51N versus BV12 was determined by one-way analysis of variance. (C) Genomic context of RSWS8N_09785 (*osp*). NCBI BLASTP and HHpred (86) analyses for homology detection were performed.

Osp is similar in structure to a SDRR, showing the typical topology (β/α)5, and it also shows the phosphorylatable aspartic residue at the end of the β3 strand (D51). However, relevant residues that are present in bona fide response regulators are missing such, as 2 acid residues (D) after the β1 strand that are required for Mg^2+^ coordination, and the conserved lysine (K) at the end of β5 are absent (33, 34) (Fig. 4A). The absence of these conserved residues suggest that this protein is not phosphorylated. In this regard, it was previously observed that *in* R. sphaeroides 2.4.1 the mutant version Osp D51A promoted the expression of the photosynthetic genes as wild-type Osp did (32).

To test if the swimming phenotype was also supported by a non-phosphorylated version of Osp, residue D51 was replaced by asparagine (N) by site-directed mutagenesis. It was previously shown that this substitution also results in a non-phosphorylatable RR, and in consequence, it cannot accomplish the role of the phosphorylated protein (35–38). The plasmid expressing the D51N version of Osp was introduced to the BV12 strain, and we observed a severe reduction in swimming, suggesting that this protein is functional in a non-phosphorylated state (Fig. 4B).

**FIG 5:**
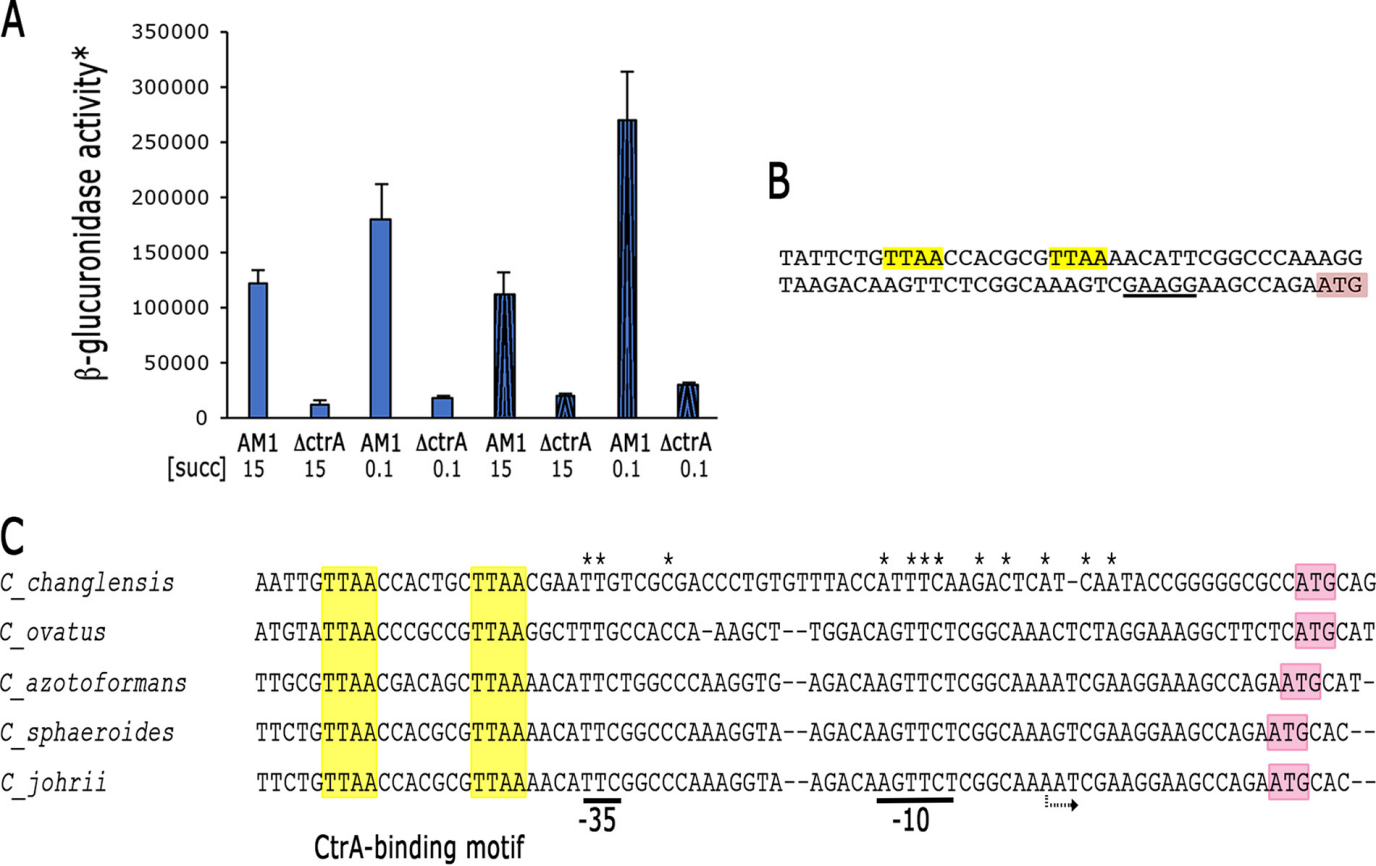
(A) β-glucuronidase activity expressed from the regulatory region of *osp* fused to the reporter gene *uidA*, in pRK415. The plasmid carrying this fusion was introduced into strains AM1 and LC7 (Δ*ctrA*::Hyg), and the amount of β-glucuronidase was determined from three independent assays. Cell extracts were obtained from cultures grown heterotrophically (solid filled columns) or photoheterotrophically (pattern filled columns) in Sistrom’s minimal medium containing 15 or 0.1 mM succinic acid as a carbon source. *Activity is expressed as picomoles of methylumbelliferone formed per minute per milligram of protein. Standard deviations are shown. A significant difference of *P < *0.01 for LC7/pRK_osp::uidA-aadA versus AM1/pRK_osp::uidA-aadA under all growth conditions was determined by one-way analysis of variance. (B) Sequence of the upstream region of *osp* showing the putative CtrA-binding site (yellow boxes). The predicted ribosome-binding site is underlined, and the start translation site is shown (pink box). (C) Alignment of the regulatory region of *osp* in the indicated organisms. The sequences matching with the consensus CtrA-binding site are highlighted in yellow. The translation codon is highlighted in pink. The conserved nucleotides after the CtrA-binding motif are indicated by an asterisk. Conserved nucleotides of the putative -35 and -10 promoter regions are underlined. The possible transcriptional start site is indicated by a curved arrow.

The *osp* gene is found 243 bp downstream of a gene encoding a putative transcriptional regulator of the TetR family, and 111 bp upstream of a gene encoding a conserved hypothetical protein (Fig. 4C). Considering the intercistronic distances between these genes, *osp* is presumably transcribed as a monocistronic mRNA. In agreement with this idea, previously reported transcriptomic data of the genes controlled by CtrA in R. sphaeroides, showed that *osp* is activated by CtrA but not the contiguous genes (29). To further support this result, a transcriptional fusion of the control region of *osp* with the reporter gene *uidA* was cloned in pRK415 and the resulting plasmid was introduced to strains AM1 and LC7 (AM1Δ*ctrA*::Hyg). As shown in Fig. 5A, very low expression of β-glucuronidase in the absence of CtrA was observed. We detected higher activities when the AM1 strain was grown photoheterotrophically and using a low concentration of succinic acid (0.1 mM) as a carbon source, a condition known to activate the CckA/CtrA system (23). As expected, in SP13 and its derivative strain BV17 (Δ*ctrA*), this plasmid promoted a low level of activity similar to that observed for the LC7 (*cckA*_L391F_ Δ*ctrA*) strain under all tested growth conditions (Fig. S1). A sequence similar to the CtrA-binding site (TAA N7 TTAA) (10, 29, 39) was identified 54 bp upstream of the start codon of Osp (ATG) (Fig. 5B). In C. crescentus, a global transcriptomic study revealed that promoters activated by CtrA have this CtrA-binding motif positioned near the -35 promoter region, considering the transcriptional start site as the +1 position (40). The regulatory regions of *osp* from species closely related to R. sphaeroides, also show the CtrA-binding motif and several conserved bases downstream that may represent the -35 and the -10 promoter regions. A conserved purine is at a proper distance to be considered the putative transcriptional start site (Fig. 5C). This conserved architecture further supports the idea that *osp* is directly activated by CtrA.

10.1128/mbio.01481-22.1 FIG S1 β-glucuronidase activity was determined using the regulatory region of *osp* fused to the reporter gene *uidA*, in pRK415 plasmid. The plasmid carrying this fusion was introduced to the indicated strains and the amount of β-glucuronidase was determined from three independent assays. This figure is similar to Fig. 5 from the main text but includes strain SP13 and BV17, both carrying the plasmid pRK_osp::uidA-aadA. Cell extracts were obtained from cultures grown heterotrophically (solid filled columns) or photoheterotrophically (pattern filled columns) in Sistrom’s minimal medium containing 15 or 0.1 mM succinic acid as a carbon source. *Activity is expressed as picomoles of methylumbelliferone formed per minute per milligram of protein. Standard deviations are shown. A significant difference of *P < *0.01 for AM1/pRK_osp::uidA-aadA versus LC7, SP13 and BV17 carrying pRK_osp::uidA-aadA was determined by one-way analysis of variance. Download FIG S1, TIF file, 2.9 MB. Copyright © 2022 Vega-Baray et al. 2022 Vega-Baray et al. https://creativecommons.org/licenses/by/4.0/ This content is distributed under the terms of the Creative Commons Attribution 4.0 International license.

### Osp inhibits CckA autophosphorylation and CtrA phosphorylation.

**FIG 6:**
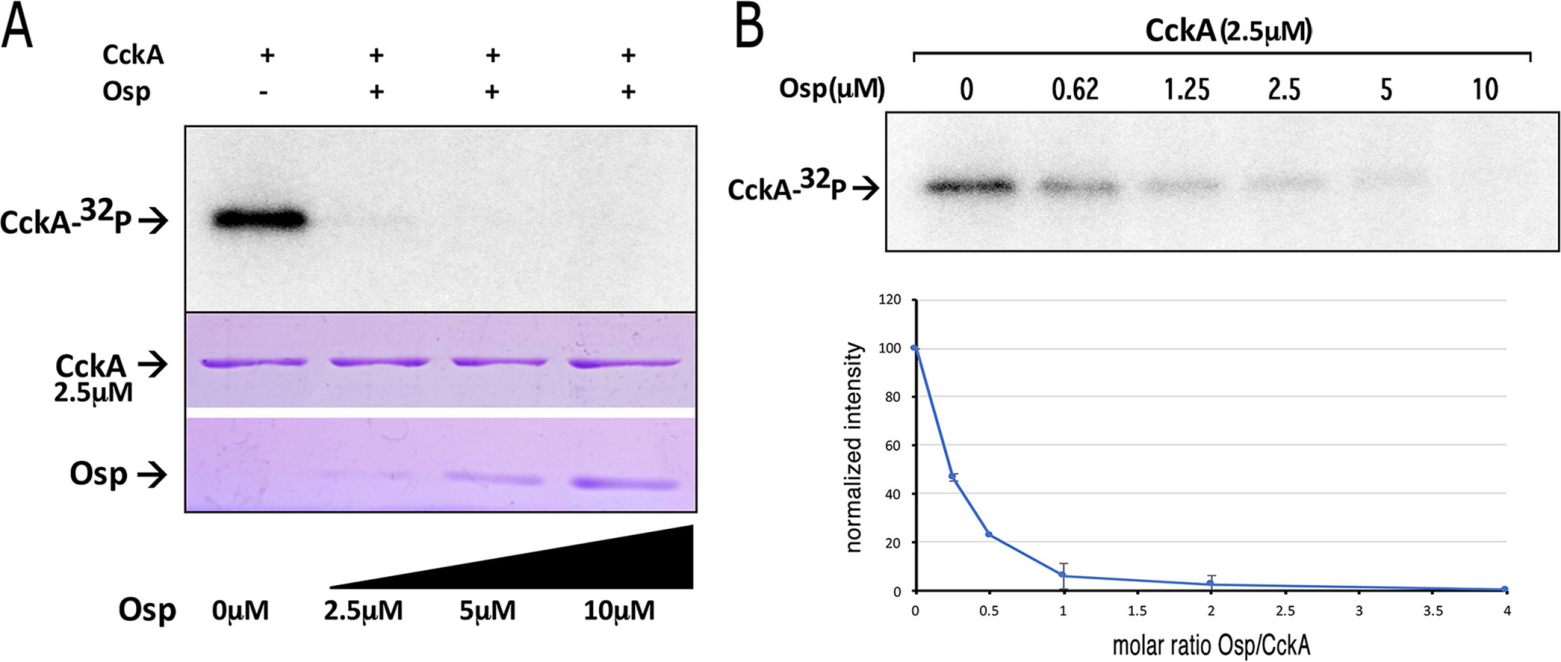
CckA phosphorylation using [γ-^32^P]ATP in the presence of different concentrations of Osp. (A) 2.5 μM CckA was incubated with increasing concentrations of Osp (0, 2.5 μM, 5 μM and 10 μM) and [γ-^32^P]ATP for 30 min and subjected to SDS-PAGE. The presence of CckA-^32^P was detected by phosphorImager visualization (upper part of the figure). The proteins used for the experiment were mixed, and an aliquot was analyzed by SDS-PAGE followed by Coomassie brilliant blue staining (shown below). (B) 2.5 μM CckA was incubated with increasing concentrations of Osp (0, 0.625 μM, 1.25 μM, 2.5 μM, 5 μM, and 10 μM) and [γ-^32^P]ATP for 30 min the mixture was subjected to SDS-PAGE. Quantification of the amount of CckA phosphorylated in the presence of the indicated concentration of Osp. The images shown correspond to representative experiments from three independent assays.

Given that Osp has a negative effect on the expression of the genes under the control of the CckA/ChpT/CtrA system, we evaluated the autophosphorylation of the cytoplasmic domain of CckA in the presence of Osp (the domain architecture of CckA is presented in Fig. 10). As shown in Fig. 6A, CckA phosphorylation is severely inhibited by the presence of Osp. It was determined that a molar ratio of 0.25 between Osp/CckA is enough to reduce CckA phosphorylation by ca. 50% (Fig. 6B), and a molar ratio of 1 practically achieved near complete inhibition. To investigate if Osp inhibits nonspecifically other HKs, we tested the kinase activity of the cytoplasmatic domain of the HK PhoR (41) in the absence and presence of Osp. This experiment showed that Osp inhibition is specific toward CckA (Fig. S2). In addition, we also demonstrated that CckA kinase activity was not affected by including in the assay a nonspecific protein containing a REC domain such as the REC domain of DctR, an active response regulator required for the transport of C4-dicarboxylic acids in R. sphaeroides (30).

10.1128/mbio.01481-22.2 FIG S2 Phosphorylation of the histidine kinases CckA and PhoR. Samples containing CckA or PhoR are indicated at the top of the image. Phosphorylation using [γ-^32^P]ATP in the presence of Osp, or the REC domain of DctR protein is indicated. A dash indicates that no other protein was added. For these assays 2.5 μM of each protein was used. The image shown corresponds to a representative experiment from three independent assays. Download FIG S2, TIF file, 1.6 MB. Copyright © 2022 Vega-Baray et al. 2022 Vega-Baray et al. https://creativecommons.org/licenses/by/4.0/ This content is distributed under the terms of the Creative Commons Attribution 4.0 International license.

10.1128/mbio.01481-22.5 FIG S5 Double yeast hybrid assay of AH109 yeast cells expressing the indicated version of Gal4BD-CckA and GAL4AD-T (SV40 T-antigen, labeled as Antig T). As shown, none of the Gal4-CckA fusions spuriously activate HIS3 and ADE2 to restore prototrophy for histidine and adenine. LW indicates that leucine (L) and tryptophan (W) are absent in the culture medium. In this medium, the presence of the plasmids is selected. LWHA indicates that leucine (L), tryptophan (W), histidine (H) and adenine (A) are absent in the culture medium. In this medium, the plasmids are selected by the absence of leucine and tryptophan, and activation of HIS3 and ADE2 is tested. Download FIG S5, TIF file, 2.7 MB. Copyright © 2022 Vega-Baray et al. 2022 Vega-Baray et al. https://creativecommons.org/licenses/by/4.0/ This content is distributed under the terms of the Creative Commons Attribution 4.0 International license.

**FIG 7:**
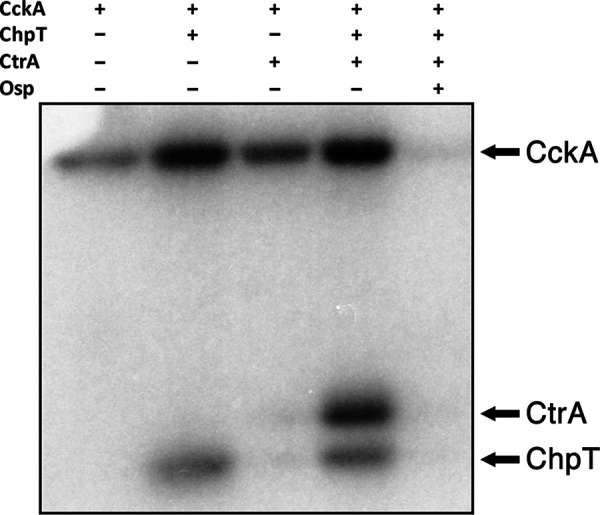
Phosphorelay reconstitution in the presence or absence of Osp. 2.5 μM of the purified components were mixed and the reaction was initiated by adding [γ-^32^P]ATP. The presence or absence of the various proteins in the reaction medium is indicated by a plus or a minus symbol. The image shown corresponds to a representative experiment from three independent assays.

In the presence of Osp, the phosphorelay from CckA to ChpT and CtrA was not observed, showing that the presence of the complete phosphorelay pathway did not affect the inhibition of CckA phosphorylation by Osp (Fig. 7). In addition, when CckA was previously phosphorylated and subsequently mixed with ChpT and CtrA or ChpT, CtrA and Osp, we did not observe a significant difference (Fig. S3), supporting the notion that Osp mainly acts by inhibiting the kinase activity of CckA.

10.1128/mbio.01481-22.3 FIG S3 Phosphotransfer reaction in the absence or presence of Osp using ^32^P-CckA. 2.5 mM of CckA was phosphorylated using [γ-^32^P]ATP as described in Material and Methods. The protein was subject to size exclusion chromatography. The elution volume was divided in two and mixed with 2.5 μM of ChpT and CtrA or 2.5 μM of ChpT, CtrA and Osp. Samples were taken at the indicated time points. 2 μL of a 1 to 100 dilution taken from the elution of volume of the size exclusion chromatography was loaded at the left in the gel to evaluate the phosphorylation of CckA (line labeled as FT). Quantitation of ^32^P-CckA is expressed as the ratio between the signal at a determined time divided by the signal at t = 0. For each point, the SD is shown. To increase the statistical accuracy of the comparison between CckA and CckA/Osp, the dephosphorylation curves were fitted to a second order model with a dummy variable and the reduced and complete models were compared. A non-significant difference (*P > *0.05) between CckA vs CckA/Osp was found. Download FIG S3, TIF file, 2.6 MB. Copyright © 2022 Vega-Baray et al. 2022 Vega-Baray et al. https://creativecommons.org/licenses/by/4.0/ This content is distributed under the terms of the Creative Commons Attribution 4.0 International license.

**FIG 8:**
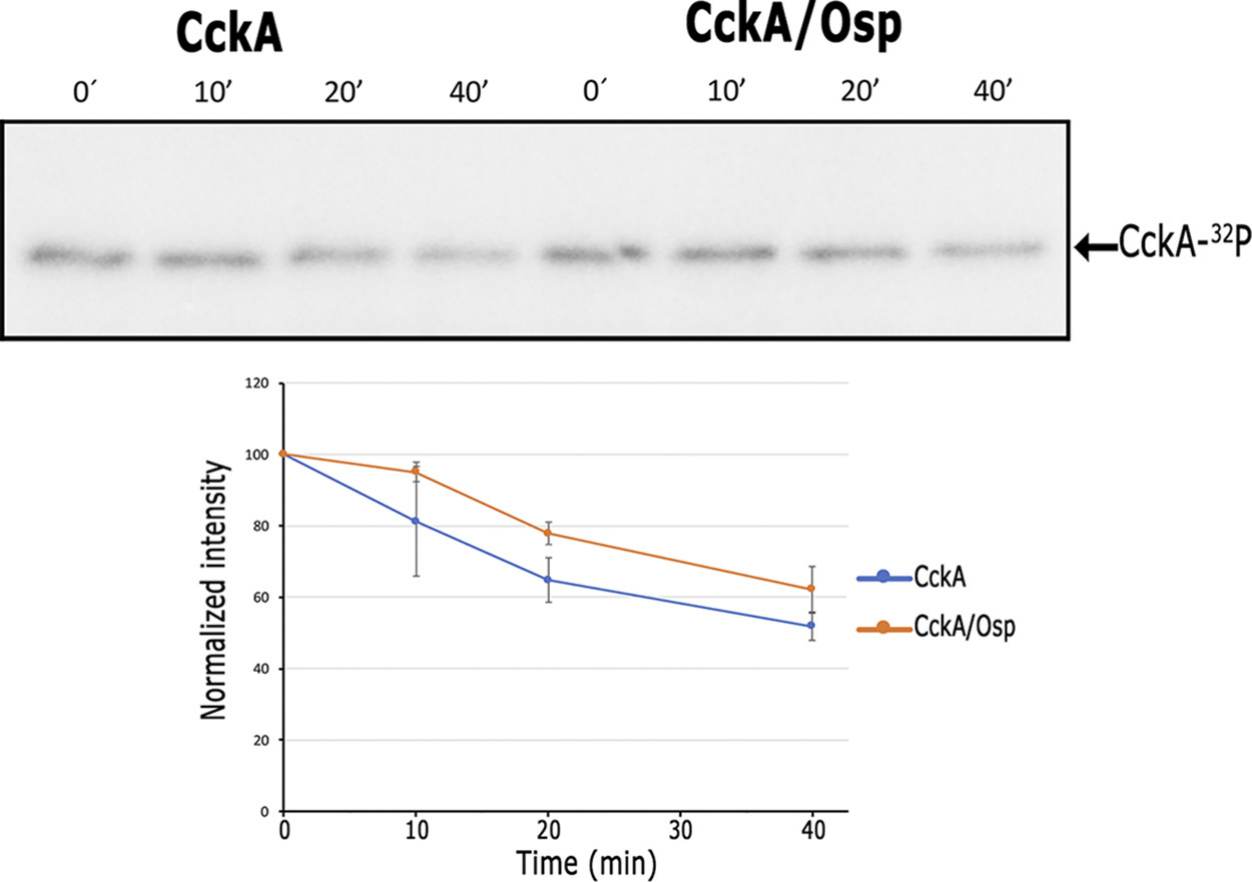
Time course assay of CckA dephosphorylation in the absence or presence of Osp. The presence of CckA-^32^P was detected by phosphorImager visualization. 2.5 μM CckA was phosphorylated and remaining ATP was removed by column-filtration chromatography, the protein was mixed with buffer or with 2.5 μM Osp, and at the indicated time points, samples were analyzed by SDS-PAGE. Quantification of CckA-P is expressed as the ratio between the signal at a determined time divided by the signal at *t* = 0. The image shown corresponds to a representative experiment from three independent assays. A non-significant difference of *P = *0.75 was determined for the slopes of the linear regression curves analyzed by a two-tailed *t* test.

We also evaluated the dephosphorylation of phospho-CckA in the presence or absence of Osp. It was observed that dephosphorylation of CckA was not affected by the equimolar presence of Osp (Fig. 8). Moreover, the addition of Osp to the previously phosphorylated proteins CckA, ChpT, and CtrA did not affect dephosphorylation of CckA (Fig. S4).

10.1128/mbio.01481-22.4 FIG S4 Phosphatase activity of the hybrid histidine kinase CckA. 2.5 μM of CckA, ChpT, and CtrA were incubated with [γ-^32^P]ATP as described in Material and Methods and after 30 min the reaction was subject to size exclusion chromatography. The elution volume was dived in two in mixed with buffer or 2.5 μM Osp. Samples were taken at the indicated times. A control reaction including only CckA and ChpT was loaded in the far-right lane in order to identify ChpT and CtrA unequivocally. Quantitation of ^32^P-CckA is expressed as the ratio between the signal at a determined time divided by the signal at t = 0. For each point, the SD is shown. A non-significant difference for CckA vs CckA/Osp was determined from the slopes of the linear regression curves using a two-tailed *t*-test. Download FIG S4, TIF file, 2.8 MB. Copyright © 2022 Vega-Baray et al. 2022 Vega-Baray et al. https://creativecommons.org/licenses/by/4.0/ This content is distributed under the terms of the Creative Commons Attribution 4.0 International license.

### CckA_L391F_ is partially resistant to Osp.

**FIG 9:**
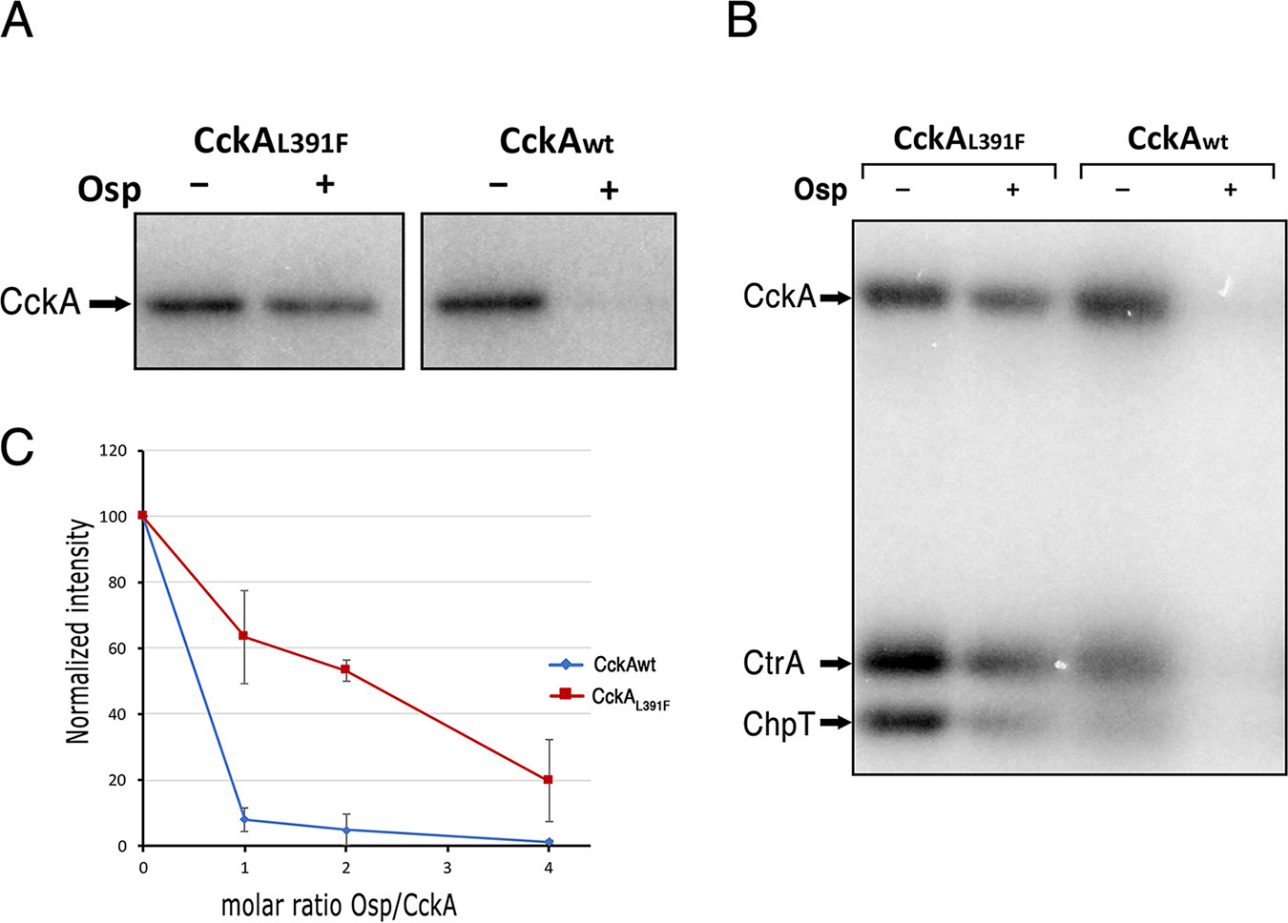
(A) Effect of the presence of Osp on the phosphorylation reaction of CckA_L391F_ and wild-type CckA. For these experiments, Osp was added at a 1:1 molar ratio to CckA. (B) Effect of Osp on the phosphorelay of the purified proteins CckA/ChpT/CtrA using CckA_L391F_ or wild-type CckA. For this experiment, 2.5 μM each protein was used. (C) 2.5 μM CckA or CckAL391F was incubated with increasing concentrations of Osp (0, 2.5 μM, 5 μM and 10 μM) and [γ-^32^P]ATP for 30 min and subjected to SDS-PAGE. Quantitation by phosphorImager analysis of the amount of phosphorylated CckA or CckAL391F in the presence of the indicated Osp/CckA molar ratio. The images shown correspond to representative experiments from three independent assays.

It was previously reported that it was possible to obtain Fla2+ strains just by the presence of a gain of function mutation in CckA, such as the one characterized for the AM1 strain i.e., CckA_L391F_ (23, 42). Nonetheless, the strong inhibition of the CckA kinase activity by Osp raised the question of how a single mutation in *cckA* can generate a Fla2+ phenotype, given that in this strain, *osp* has a wild-type sequence. Therefore, it follows that CckA_L391F_ must be somewhat refractory to the action of Osp. To test this possibility, we carried out a phosphorylation assay using CckA_L391F_ and an equimolar concentration of Osp. As shown in Fig. 9A, phosphorylation of CckA_L391F_ is still detectable as compared with wild-type CckA. As expected from this result, in the presence of Osp, CtrA-P was still clearly detected when CckA_L391F_ was used in the phosphorelay assay (Fig. 9B). To evaluate if inhibition of CckA_L391F_ could require a higher concentration of Osp, increased amounts of this protein were included in the phosphorylation assay. This experiment revealed that inhibition of CckA_L391F_ required a concentration approximately four times higher of Osp than that required to inhibit wild-type CckA (Fig. 9C).

### Osp interacts with the transmitter domain of CckA.

**FIG 10:**
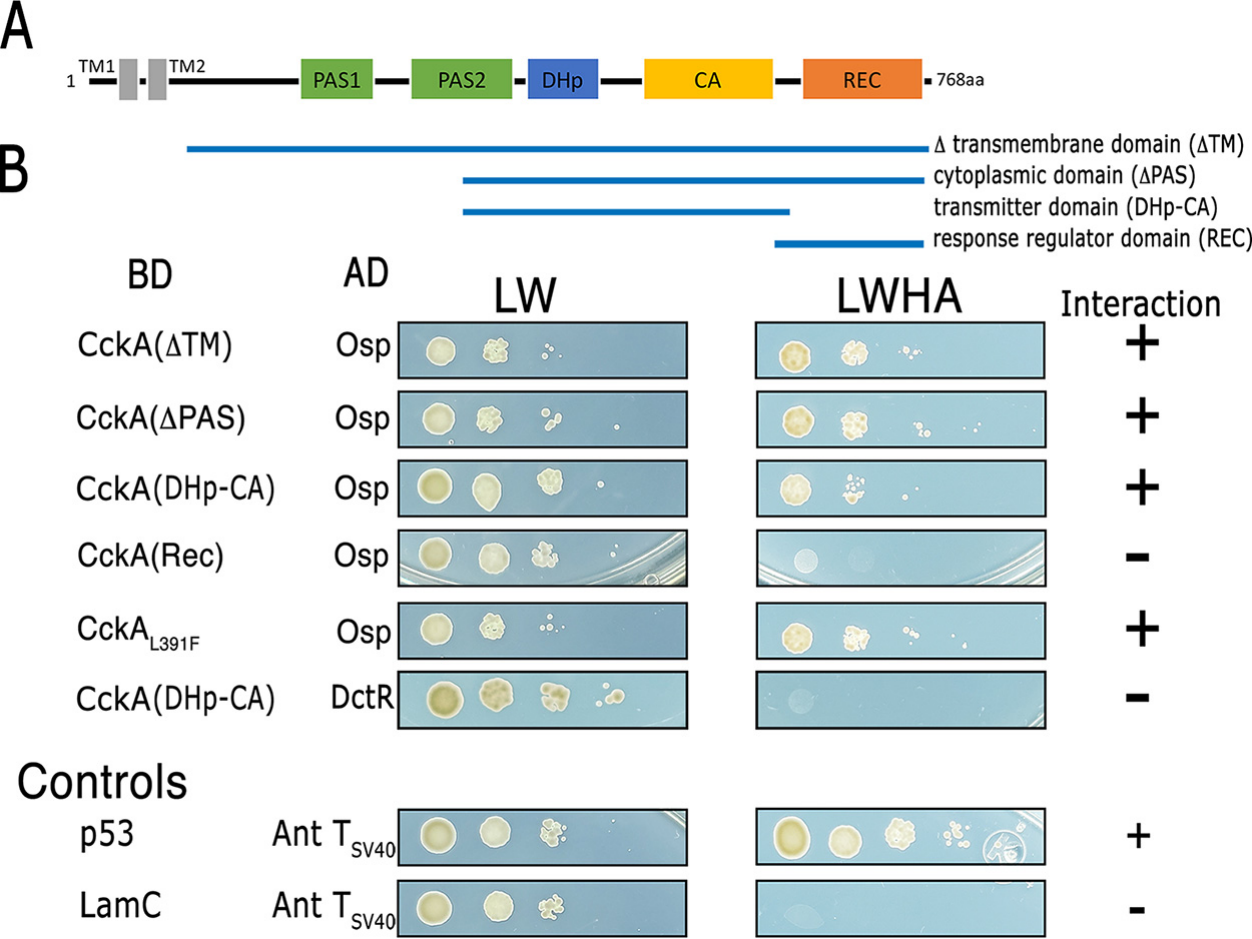
(A) Domain architecture of CckA, the domains present in each construct are indicated below. (B) Interaction of Osp with CckA tested by the yeast double hybrid assay. Yeast cells were transformed with the pair of plasmids carrying the DNA binding domain of GAL4 (BD) fused to a CckA domain, and the activation domain (AD) of GAL4 fused to Osp. Under the column labeled BD, the CckA domain cloned in pGBKT7 plasmid is indicated. Protein-protein interactions were evaluated by testing histidine (H) and adenine (A) prototrophy. The letters L, W, H and A indicate the nutrient that is absent in the culture medium. LW indicates the absence of leucine and tryptophan in the culture medium. LWHA indicates the absence of leucine, tryptophan, histidine, and adenine. Positive and negative interactions between Osp and CckA are summarized at the far-right. Below the positive and negative interaction controls represented by GAL4AD-T (simian virus 40 large antigen T) and GAL4BD-Lam (lamin C) (−), and GAL4AD-T and GAL4BD-p53 (+) pairs are shown. The control experiments using AH109 yeast cells expressing the different versions of Gal4BD-CckA and GAL4AD-T (SV40 T-antigen) are shown in Fig. S5.

To obtain evidence of the physical interaction between CckA and Osp, we carried out a yeast double hybrid assay using different domains of CckA fused to the DNA binding domain (BD domain) of the transcriptional activator Gal4, whereas Osp was fused to the activation domain (AD domain) of Gal4. In this assay, a positive interaction between the proteins to be tested brings together the AD and the BD domains of Gal4 creating a functional activator that promotes expression of HIS3 and ADE2. It has been reported that HIS3 has a leaky expression (43); therefore, testing the expression of HIS3 and ADE2 simultaneously reliably indicates a strong interaction between the tested proteins (*idem*). In the yeast strain AH109, the absence of leucine (L) and tryptophan (W) selects the presence of the plasmids encoding the fusion proteins, and the expression of the reporter genes HIS3 and ADE2 is detected by histidine (H) and adenine (A) prototrophy. The experiments showed a robust growth on plates without histidine and adenine (LWHA) for cells expressing Osp and CckA protein fusions, indicating a strong interaction between these proteins. This interaction is mediated by the transmitter domain of CckA given that the PAS and the REC domains were dispensable (Fig. 10).

It should be noted that the binding assay did not reveal significant differences between the interactions of Osp with CckA or with CckA_L391F_, suggesting that either the assay is not sensitive enough to differentiate in affinity or that the CckA_L391F_ mutation does not interfere with Osp binding but makes the protein less sensitive to its inhibitory effect.

To explore if these results could be explained by an unspecific interaction between CckA and any protein containing a REC domain, we fused the AD domain of GAL4 to the REC domain of the transcriptional activator DctR. We did not detect interaction between CckA and the REC domain of DctR supporting the idea of a specific interaction between the transmitter domain of CckA and Osp (Fig. 10). A slight growth in the absence of H and A was detected for the cells expressing CckA (REC) and Osp (Fig. 10). However, a low level of auto-activation promoted by pGBKT7_cckA-REC could explain this residual growth (Fig. S5). The expression of the fusion proteins in these experiments was confirmed by Western blotting (data not shown).

**FIG 11:**
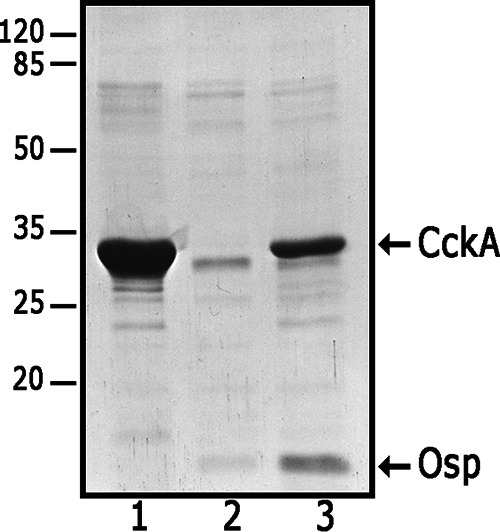
*In vivo* pull-down of Osp using His_6_-CckA. A cell extract obtained from E. coli cells over-expressing only His_6_-CckA (lane 1); only Osp without a His6X-tag (lane 2); or simultaneously both proteins i.e., His_6_-CckA and Osp without a His6X-tag (lane 3) were used to purify His_6_-CckA by affinity chromatography using Ni-NTA-agarose. The purified proteins were subjected to SDS-PAGE and visualized by Coomassie brilliant blue staining. It should be stressed that the over-expression of these proteins was carried out using the T*7* promoter cloned upstream of each gene. Migration of the molecular mass markers is shown at the left and expressed in kDa.

The interaction between CckA and Osp was further corroborated by the co-purification of a non-tagged version of Osp along with His_6_-CckA by Ni-NTA affinity chromatography (Fig. 11).

### Accumulation of Osp does not mirror its transcriptional expression pattern.

**FIG 12:**
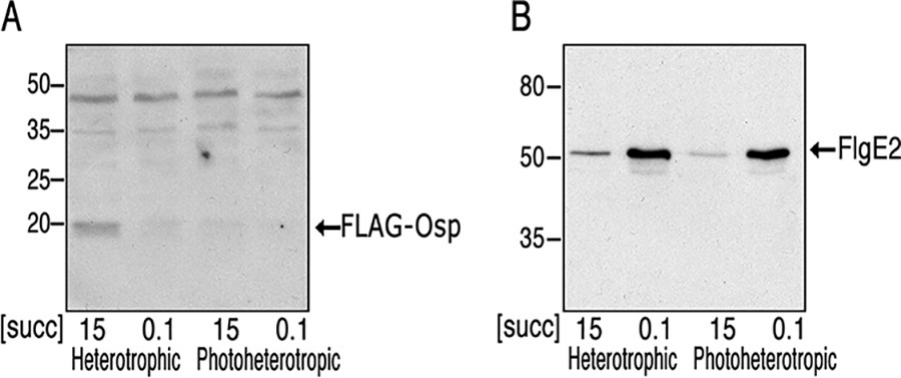
Total cell extracts obtained from BV18 cells grown under the indicated conditions, were subjected to SDS-PAGE and tested by Western blotting analysis using anti-FLAG (A) and anti-FlgE (B) antibodies. The growth condition, and the concentration of succinic acid (mM) used as carbon source is indicated below. The migration of the molecular mass markers (kDa) is indicated at the left.

The expression of *osp* is activated by CtrA, and Osp inhibits the kinase activity of CckA, creating a negative feedback loop. However, previous evidence suggests that *osp* is expressed at low levels even in the absence of CtrA (29). These observations raise the question of how the TCS CckA/ChpT/CtrA reaches a high level of activity. The simplest solution would be to avoid Osp accumulation when the TCS needs to be activated. Therefore, we tested if the presence of Osp in total cell extracts mirrors its transcription profile. To reveal the presence of Osp, we carried out a Western blot analysis using an anti-FLAG antibody that allowed us to recognize an N-terminal tagged version of Osp. Importantly this version of the protein that is fairly functional (Fig. S6) is expressed from its native position in the chromosome and uses the same translational start site of Osp. As shown in Fig. 12A, Osp was strongly detected in cells grown aerobically in 15 mM succinic acid, but it was barely detected in cells grown in 0.1 mM succinic acid and under photoheterotrophic growth, irrespectively of the succinic acid concentration. As a control of the CckA/ChpT/CtrA TCS activity, the same samples were tested with an antibody that recognizes the flagellar hook protein FlgE2. As expected for a protein encoded by a gene controlled by CtrA-P, FlgE2 was clearly detected when the cells were grown in 0.1 mM succinic acid and severely reduced in 15 mM (Fig. 12B). In general, this result reveals that the presence of Osp does not follow the same pattern to that observed for a CtrA-activated gene, suggesting that Osp stability could be regulated to maintain a low level when the TCS CckA/ChpT CtrA is activated.

10.1128/mbio.01481-22.6 FIG S6 Swimming phenotype of BV19 strain expressing FLAG-Osp. Plates containing Sistrom’s minimal medium supplemented with 0.1 mM succinic acid were inoculated with 2 μL of a saturated culture of the indicated strain. Plates were incubated for 60 h. The diameter of the swimming ring was determined from at least three independent experiments, AM1 = 1.69 cm SD ± 0.9; SP13 = 1.06 cm SD ± 0.1; BV11 = 2.8 cm SD ± 0.01; BV19 = 1.7 cm SD ± 0.05. A significant difference of *P < *0.01 for BV19 versus BV11 and SP13 was determined by one-way analysis of variance. Download FIG S6, TIF file, 2.4 MB. Copyright © 2022 Vega-Baray et al. 2022 Vega-Baray et al. https://creativecommons.org/licenses/by/4.0/ This content is distributed under the terms of the Creative Commons Attribution 4.0 International license.

### Osp is conserved in specific clades of the *Rhodobacterales*.

**FIG 13:**
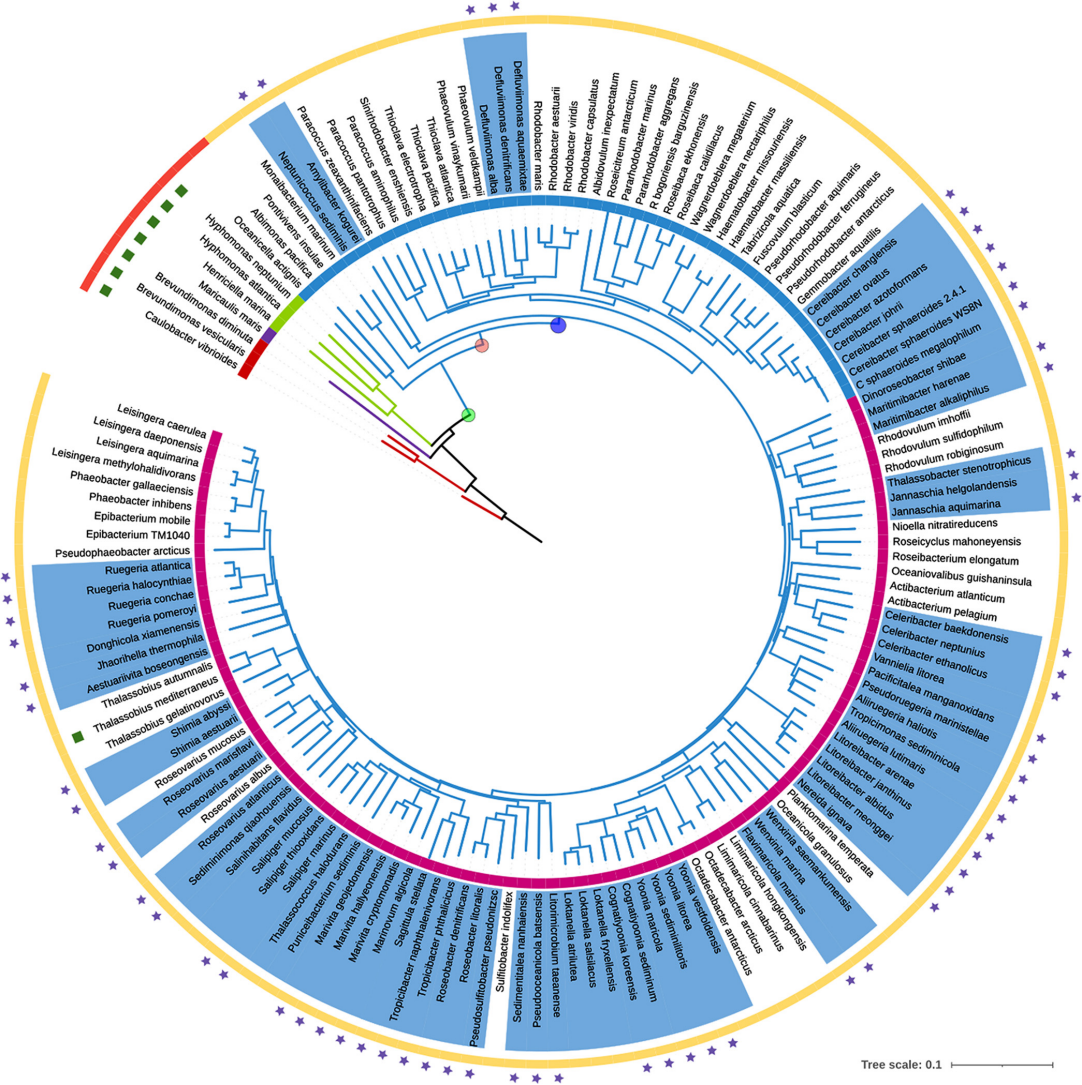
Phylogenetic distribution of *osp* in *Rhodobacterales*. Species phylogeny based on RpoC, the tree was generated by the neighbor joining method using clustal simple phylogeny and edited with iTOL. From inside to outside, color of the branches indicates the Order: *Caulobacterales* (red), *Maricaulales* (purple); *Hyphomonadales* (green), *Rhodobacterales* (blue). Circle below the species name indicates the Family: *Caulobacteraceae* (red), *Maricaulaceae* (purple), *Hyphomonadaceae* (green), *Rodobacteraceae* (blue), and *Roseobacteraceae* (magenta). In the circle depicting the species names, the presence of Osp is indicated by a blue background. The presence of DivK is represented by green squares above the species names, the presence of a long DivL (red) or a short DivL (yellow). The stars represent the presence of a putative CtrA-binding site in the regulatory region of *osp*. Green dot possible point of DivK loss, pink dot truncation of DivL (short DivL), blue dot represents earliest possible appearance of Osp. Complete information for each species, the GenBank accession number for each genome, the accession number for RpoC, Osp, and DivK are included in Table S2.

The TCS CckA/ChpT/CtrA is present in alphaproteobacteria but the regulatory proteins that control this system show adaptations in specific clades of this group (4). A search for the *osp* gene in different bacteria revealed its presence in species closely related to Rhodobacter sphaeroides (now *Cereibacter sphaeroides*) and in species of the *Defluviimonas* genus. However, it is conspicuously absent in other species within the *Rhodobacteraceae* family. In contrast, *osp* is widely distributed in species of the *Roseobacteraceae* family (Fig. 13, species with Osp are shaded in blue), and it was not found in any other genera of alphaproteobacteria. Interestingly, the presence of Osp in Amylibacter kogurei that diverges before the division between *Rhodobacteraceae* and *Roseobacteraceae* and the narrow distribution of the *osp* gene in the *Rhodobacteraceae* family, suggests a complex evolution in which the gene could have been lost in several genera of this family and retained only in a few of them i.e., *Cereibacter* and *Defluviimonas*. A more consistent distribution of *osp* among the different genera of the *Roseobacteraceae* family was observed.

10.1128/mbio.01481-22.9 TABLE S2 Species and accession numbers of the sequences used to obtain the phylogenetic tree. Download Table S2, DOCX file, 0.04 MB. Copyright © 2022 Vega-Baray et al. 2022 Vega-Baray et al. https://creativecommons.org/licenses/by/4.0/ This content is distributed under the terms of the Creative Commons Attribution 4.0 International license.

Interestingly, for many of these bacterial species, we found a sequence similar to the CtrA-binding site in the regulatory region of *osp* (Fig. 13, purple stars and Fig. S7). If these sites are functional, the regulatory circuit that controls the TCS CckA/ChpT/CtrA in these bacterial species will also include Osp, probably creating a negative feedback loop of the system as occurs in R. sphaeroides.

10.1128/mbio.01481-22.7 FIG S7 Analysis of the intergenic upstream regions of the *osp* orthologs using MEME. (A) The red boxes represent the best motif found by MEME with the consensus TTAACCHTTYGTTAA and their respective e-values for each sequence. (B) MEME results showing at the far-right column, the distance between the motif and the ATG start codon of *osp.* Download FIG S7, DOCX file, 0.4 MB. Copyright © 2022 Vega-Baray et al. 2022 Vega-Baray et al. https://creativecommons.org/licenses/by/4.0/ This content is distributed under the terms of the Creative Commons Attribution 4.0 International license.

As reported, a short version of the DivL that lacks the pseudo-histidine kinase domain and the absence of its interacting partner, the protein DivK, are a common trait in *Rhodobacterales* (4, 44, 45) (Fig. 13).

Noteworthy, we found a possible progression of events that seem to have preceded the appearance of Osp. Initially, following the loss of DivK (Fig. 13), we observed two versions of DivL i.e., in the group represented by Oceanicella actignis and Albimonas pacifica, we detected the presence of a large version of DivL, although its HK and CA domains were degenerated but still identified in a bioinformatic prediction, and for the rest of the *Rhodobacterales*, we detected the presence of a short version of DivL. After the loss of DivK and truncation of DivL, Osp could have appeared early (Fig. 13, purple dot) and subsequently be lost, or could have appeared at a later point and horizontally acquired by other species.

Interestingly, from this analysis, a possible horizontal transfer was detected in Thalassobius mediterraneum (*Roseobacteraceae*) in which the short version of DivL was present but, in contrast to other related bacteria, DivK was also present. The presence of DivK in this microorganism seems to be the product of a horizontal transfer event given that no other species in the Order have DivK. Comparison of DivK of T. mediterraneum by BLAST showed that DivK from Henricella marina (*Hypomonadaceae*) is the most similar sequence, suggesting that this may be the possible origin of the gene. Furthermore, in these organisms *divK* is found upstream of *pleD* and PleD from H. marina is also the best hit of PleD from T. mediterraneum.

## DISCUSSION

The negative control of TCSs in bacteria is frequently carried out by diverse proteins that modulate HKs. For instance, in Escherichia coli, the HK NtrB (NRII) is switched to its phosphatase state upon binding of the sensor protein PII (46, 47). In Bacillus subtilis, the HK KinA is inhibited by SdA and KipI (48, 49). These inhibitory proteins do not share structural similarity, but all of them bind to the transmitter domain of their cognate HK (49–53). Regarding its structure, PII is a β-α-β homotrimer (54); in contrast, Sda is a 46-residue protein that adopts an antiparallel hairpin structure (55, 56), and the 240 residues long KipI belongs to the cyclophilin-like domain superfamily (57, 58). These examples illustrate that, often, inhibition of the HKs is accomplished by structurally unrelated proteins.

FixT, a SDRR has been reported to act as a negative regulator of the SHK FixL in Sinorhizobium meliloti and C. crescentus (59–61). It was demonstrated that FixT inhibits autophosphorylation of FixL without affecting its dephosphorylation rate (60, 62). Given that phosphorylation of FixT was not observed, it was ruled out that it could act as a phosphate sink (60, 62).

In this study, we showed that Osp a SDRR, is responsible of inhibiting the TCS CckA/ChpT/CtrA in R. sphaeroides. An inactivating mutation in *osp* causes the expression of the CtrA-activated genes, such as the flagellar and chemotactic genes. Consistent with this result, *in vitro* phosphorylation assays showed that Osp directly inhibits phosphorylation of the HHK CckA, whereas its dephosphorylation rate is not affected. The presence of ChpT and CtrA did not relieve this inhibition and phosphorylation of Osp was not observed.

The effect of FixT and Osp indicate that inhibition of HKs by SDRRs could be a common mechanism and that it can also be implicated in regulating HHKs that are structurally more complex than canonical HKs.

We also determined that Osp interacts with the transmitter domain of CckA. Since Osp is a RR and it interacts with the transmitter domain of CckA, it is tempting to propose that the interaction between these two proteins occurs through the same protein regions that mediate the interaction between other HKs and their cognate RRs. If this is the case, binding of Osp may then interfere with the access of the CA domain to the phosphorylatable His residue. Furthemore, we observed that Osp was able to inhibit CckA autophosphorylation when present in a substoichiometric ratio, raising the possibility that Osp could bind a CckA dimer and cause a conformational change that would prevent its phosphorylation. In this regard, it should be stressed that most of the SHK commonly exists as dimers and structural studies support the view that, in the autokinase state, the packing of the DHp helixes is different for each monomer and, in consequence, only one CA domain is found in close proximity to the phosphorylatable histidine (63–65). It is possible that Osp could have a higher affinity for this protomer and, from this position, it could prevent the rearrangement of the other protomer. Determination of the crystal structure of the complex would help to clarify the stoichiometry of the complex.

We observed that Osp was not phosphorylated; we believe that the absence of two acid residues before the α-helix 1 could account for this. The lack of these acid residues is also observed for the FixT proteins of S. meliloti and C. crescentus, neither of which are phosphorylated (60, 62).

Regarding the transcriptional control of *osp*, a global transcriptomic analysis of the CtrA-dependent genes revealed that *osp* transcription is activated by CtrA but in its absence, *osp* expression is still detectable (29). We confirmed this result using a transcriptional fusion of the regulatory region of *osp* to the *uidA* reporter gene. Transcriptional activation of *osp* by CtrA generates a negative feedback loop that limits the activity of the system; the basal expression of *osp* will maintain the system inactive until an unknown signal promotes Osp degradation. This postranscriptional control may be enough to turn on and keep the system active. Increasing the expression of *osp* from a plasmid significantly reduced the swimming ability of the AM1 strain and, therefore, we presume that a precise balance of Osp concentration, based on transcriptional and posttranslational mechanisms, is essential to control the TCS CckA/ChpT/CtrA in R. sphaeroides.

Proteolysis of FixT and Sda is also the release mechanism of their respective SHK from inhibition (62, 66), showing that this molecular strategy is commonly used to reduce the intracellular amount of an inhibitor.

The presence of Osp in many different genera of bacteria that lack the DivK/DivL system suggests that a negative control of the TCS CckA/ChpT/CtrA must be physiologically important. In these cases, Osp would accomplish an analogous role to that of DivK-P of C. crescentus. In bacteria where DivK and DivL have been characterized, such as in C. crescentus and in a few species of *Rhizobiales*, the TCS CckA/ChpT/CtrA is essential, and its activity is modulated by different proteins that are mainly controlled by internal cues, such as DNA replication or cell division (5, 67–69). In this context, Osp represents a powerful alternative solution that could make the system sensitive to environmental signals.

## MATERIALS AND METHODS

### Bacterial strains and growth conditions.

The strains used in this work are listed in Table 1. R. sphaeroides strains were grown in Sistrom’s minimal medium (70), without cas amino acids and supplemented with 15 mM or 0.1 mM succinic acid as carbon source. Growth conditions were reported previously (29). Saccharomyces cerevisiae was grown at 30°C in YPDA or in synthetic defined minimal medium (Clontech).

**TABLE 1:** Strains and plasmids

Strain or plasmid	Description	Source
Rhodobacter sphaeroides strains		
AM1	SP13 derivative; Δ*fleQ*::Kan, *cckA*L391F	27
BV6	SP20 derivative; Δ*fliF1*::*aadA, osp*H115D	This study
BV7	SP13 derivative; Δ*fleQ*::Kan, *osp*77aa	This study
BV8	SP20 derivative; Δ*fliF1*::*aadA*, *osp*AV+	This study
BV9	SP13 derivative; Δ*fleQ*::Kan, *osp*11shift	This study
BV10	SP20 derivative; Δ*fliF1*::*aadA*, Δ*osp*::Hyg	This study
BV11	SP13 derivative; Δ*fleQ*::Kan, Δ*osp*::Hyg	This study
BV12	AM1 derivative; Δ*fleQ*::Kan, *cckA*L391F, Δ*osp*::Hyg	This study
BV13	SP13 derivative; Δ*fleQ*::Kan, Δ*osp*::Hyg, Δ*ctrA*::*aadA*	This study
BV14	BV11 derivative; Δ*ctrA*::*aadA*	This study
BV15	BV11 derivative; Δ*chpT*::*aadA*	This study
BV16	BV6 derivative; Δ*cckA*::Hyg	This study
BV17	SP13 derivative; Δ*ctrA*::Hyg	This study
BV18	AM1 derivative; FLAG-*osp*	This study
BV19	SP13 derivative; FLAG-*osp*	This study
JHV3	AM1 derivative; Δ*fleQ*::Kan, *cckA*L391F, *mcpB*::*uidA*-*aadA*	29
LC7	AM1 derivative; Δ*fleQ*::Kan, *cckA*L391F, Δ*ctrA*::Hyg	Laboratory collection
SP13	WS8N derivative; Δ*fleQ*::Kan	25
SP20	WS8N derivative; Δ*fliF1*::*aadA*	Laboratory collection
Escherichia coli strains		
LMG194	Protein expression strain	Invitrogen
TOP10	Cloning strain	Invitrogen
Rosetta	Protein expression strain	Novagen
Yeast strains		
AH109	Reporter strain for two-hybrid screening *HIS3*, *ADE2,* and *lacZ*	Clontech
Plasmids		
pBAD HisB	Expression vector of His6X-tagged proteins; Ap	Invitrogen
pBAD_chpT	pBAD/HisB expressing His6-ChpT	Laboratory collection
pBAD_ctrA	pBAD/HisA expressing His6-CtrA	87
pBAD/His-CckA	pBAD/HisB expressing the cytoplasmic domain of CckA fused to His6x	23
pBAD/His-CckA L391F	pBAD/HisB expressing the cytoplasmic domain of CckAL391F fused to His6x	23
pBADHis-dctR	pBAD-His expressing DctR fused to His6x	30
pET28a	Expression vector for His6x-tagged proteins, Kan	Novagen
pET28A_6XHis-cckA_osp	pET28 expressing the transmitter domain of CckA fused to His6x, and Osp	This study
pET28A_6xHis-cckA	pET28 expressing the transmitter domain of CckA fused to His6x	This study
pET28a_His6x-PhoR	pET28a expressing the cytoplasmic domain of PhoR fused to His6x	This study
pET28a_osp	pET28a expressing Osp	This study
pET28a_osp6xHis	pET28a expressing Osp fused to His6x	This study
pGADT7	Plasmid for double hybrid assay with the Gal4 activation domain *LEU2*	Clontech
pGADT7_osp	pGADT7 expressing the fusion Gal4AD-Osp	This study
pGADT7_REC-DctR	pGADT7 expressing the fusion Gal4AD-REC-DctR	This study
pGBKT7	Plasmid for double hybrid assay with the Gal4 DNA binding domain *TRP1*	Clontech
pGBKT7_cckA_DHp-CA	pGBKT7 expressing the fusion of GAL4AD-CckA DHp domain	This study
pGBKT7_cckA-REC	pGBKT7 expressing the fusion of Gal4AD-CckA-REC	This study
pGBKT7_cckADPas	pGBKT7 expressing the fusion Gal4BD-CckAΔPAS	This study
pGBKT7_cckAΔTM	pGBKT7 expressing the fusion Gal4BD-CckAΔTM	This study
pIJ963	Plasmid source of the Hyg cassette	88
pJQ200mp18	Suicide vector for R. sphaeroides	89
pJQ200_Δosp::Hyg	pJQ200mp18 carrying Δ*osp*::Hyg	This study
pRK415	Expression vector used in R. sphaeroides, Tc	90
pRK_osp	pRK415 expressing Osp	This study
pRK_osp D51N	pRK415 expressing Osp D51N	This study
pRK_osp::uidA-aadA	pRK415 arring the transcriptional fusion *osp*-*uidA*	This study
pSUP11	Plasmid for epitope tagging	91
pTZ18R_Δosp::Hyg	pTZ18R carrying Δ*osp*::Hyg	This study
pTZ18R_ospUPDW	pTZ18R carrying the upstream and downstream regions of osp	This study
pTZ18R/19R	Cloning vectors, Ap	92
pTZ19R Bam-	pTZ19R without BamHI site	Laboratory collection
pTZospFLAG_1.7	pTZ19RBamHI- containing the upstream and coding region of FLAG-osp	This study
pWM5	Vector source of the *uidA*-*aadA* cassette	93

### Molecular biology techniques.

Standard methods were used to obtain chromosomal or plasmid DNA (71). DNA was amplified with the appropriate oligonucleotides (Table S1) using Prime Star *Taq* DNA polymerase (TaKaRa) according to the recommendations of the manufacturer. Standard methods were used for transformation, ligation, and other related techniques.

10.1128/mbio.01481-22.8 TABLE S1 Oligonucleotides used in this work. Download Table S1, DOCX file, 0.1 MB. Copyright © 2022 Vega-Baray et al. 2022 Vega-Baray et al. https://creativecommons.org/licenses/by/4.0/ This content is distributed under the terms of the Creative Commons Attribution 4.0 International license.

### Motility assays.

Motility was tested in soft agar plates (0.22%) as described previously (29).

### Genome sequence of BV6 and analysis.

A genomic library was constructed and subjected to 2 × 76-bp pair-end sequencing on the Illumina NexSeq 500 platform. Reads were mapped against the genome of R. sphaeroides WS8N using bowtie2 (72). A BCF file was created using SAMtools, variants (SNPs and indels) were called using BCF tools (73, 74). Changes were confirmed by PCR followed by Sanger sequencing.

### β-glucuronidase assay.

Enzymatic activities were performed following previously reported protocols (29, 75). Protein content was determined with a Bio-Rad protein assay kit.

### Strains isolated in this work.

The strains isolated for this work were obtained following the procedures described in the supplementary methods (Text S1).

### Protein overexpression and purification.

Proteins were overexpressed and purified using standard methods (23, 76). Details of the procedures are described in supplementary methods (Text S1).

### Phosphorylation reactions.

His_6_-CckA or the mutant version, His_6_-CckA_L391F_, was adjusted to 2.5 μM in HEPES buffer (33 mM HEPES, 10 mM MgCl_2_, 50 mM KCl, 1 mM dithiothreitol, and 10% glycerol pH 7.5). Osp-His_6_ was added as required at the indicated concentration. The phosphorylation reaction was started by adding 500 μM ATP with 1 μL of [γ-^32^P]ATP to a final volume of 30 μL. At the desired time points, a sample of 5 μL was withdrawn, and the reaction was stopped by the addition of 5 μL of Laemmli sample buffer (4X) (77). After SDS-PAGE, radioactivity was visualized and quantified using phosphorimaging screens. The phosphotransfer reactions were performed by mixing His_6_-CckA, or His_6_-CckA and 2.5 μM Osp-His_6_, together with purified His_6_-ChpT (2.5 μM), and His_6_-CtrA (2.5 μM) in HEPES buffer. The phosphorylation reaction was started by adding 500 μM ATP with 1 μL of [γ-^32^P]ATP to a final volume of 30 μL. After 20 min the reaction was stopped by the addition of 30 μL of Laemmli sample buffer (4X). Alternatively, for the experiment shown in Fig. S3, 2.5 μM CckA was phosphorylated with [γ-^32^P]ATP for 30 min and subject to size exclusion chromatography. The elution volume was divided in two and mixed with 2.5 μM ChpT and CtrA or with 2.5 μM ChpT, CtrA, and Osp. After mixing, samples were taken at the indicated times and subjected to SDS-PAGE.

### Phosphatase activity of CckA.

2.5 μM His_6_-CckA was phosphorylated using [γ-^32^P]ATP for 20 min. After this time, the remaining ATP was removed by size exclusion chromatography. The elution volume of 40 μL was divided in two and mixed either with buffer or with 2.5 μM Osp-His_6_ to a final volume of 30 μL. After mixing, samples were taken every 10 min and the reaction was stopped using 5 μL of Laemmli sample buffer (4X). Alternatively, phosphatase activity was also evaluated, initiating the reaction with 2.5 μM CckA, ChpT, and CtrA, previously phosphorylated with [γ-^32^P]ATP for 30 min and subject to size exclusion chromatography. The elution volume was divided in two and mixed either with buffer or 2.5 μM Osp-His_6_. Samples were taken at the indicated times and analyzed by SDS-PAGE.

### Yeast double hybrid assays.

Protein interactions were tested using the Matchmaker GAL4 system 3 following the instructions of the manufacturer (Clontech).

### Western blot.

Total cell extracts were subjected to SDS-PAGE (77). Proteins were transferred onto a nitrocellulose membrane and probed using anti-FLAG (1:10,000), anti-FlgE2, or anti FliA (1:30,000) immunoglobulins (78, 79). Detection was done with a secondary antibody conjugated to alkaline phosphatase and developed with CDPStar (Applied Biosystems).

### Phylogenetic analysis.

The *Rhodobacterales* species were selected if their genomes were complete or nearly complete >95% with low level of contamination <5% as outlined in CheckM (80). The RpoC protein was identified by BLASTP. The RpoC proteins were aligned with Muscle version 3.8 (81). The phylogenetic tree was constructed by neighbor joining method (82).

### Bioinformatic analysis of the sequences.

For each genome in Fig. 13, the intergenic region between *osp* and the upstream gene was obtained from the NCBI database. These sequences were searched for the presence of DNA motifs using MEME version 5.4.1 (83). For analysis of the sequences in Fig. 4, secondary structure predictions were carried out using Psipred (84) and protein homology was evaluated using Swiss-Model (85) and the crystal structure of CheY (PDB 6TG7).

10.1128/mbio.01481-22.10 TEXT S1 Supplementary methods. Includes the following sections: Strains isolated in this work, Protein overexpression and purification, Protein copurification, and References. Download Text S1, DOCX file, 0.03 MB. Copyright © 2022 Vega-Baray et al. 2022 Vega-Baray et al. https://creativecommons.org/licenses/by/4.0/ This content is distributed under the terms of the Creative Commons Attribution 4.0 International license.
