# Supplementary figures and images for: The Histidine Kinase CckA Is Directly Inhibited by a Response Regulator-like Protein in a Negative Feedback Loop
Source: mBio. 2022 Jul 25;13(4):e01481-22. doi: 10.1128/mbio.01481-22 (PMC9430884; doi:10.1128/mbio.01481-22)

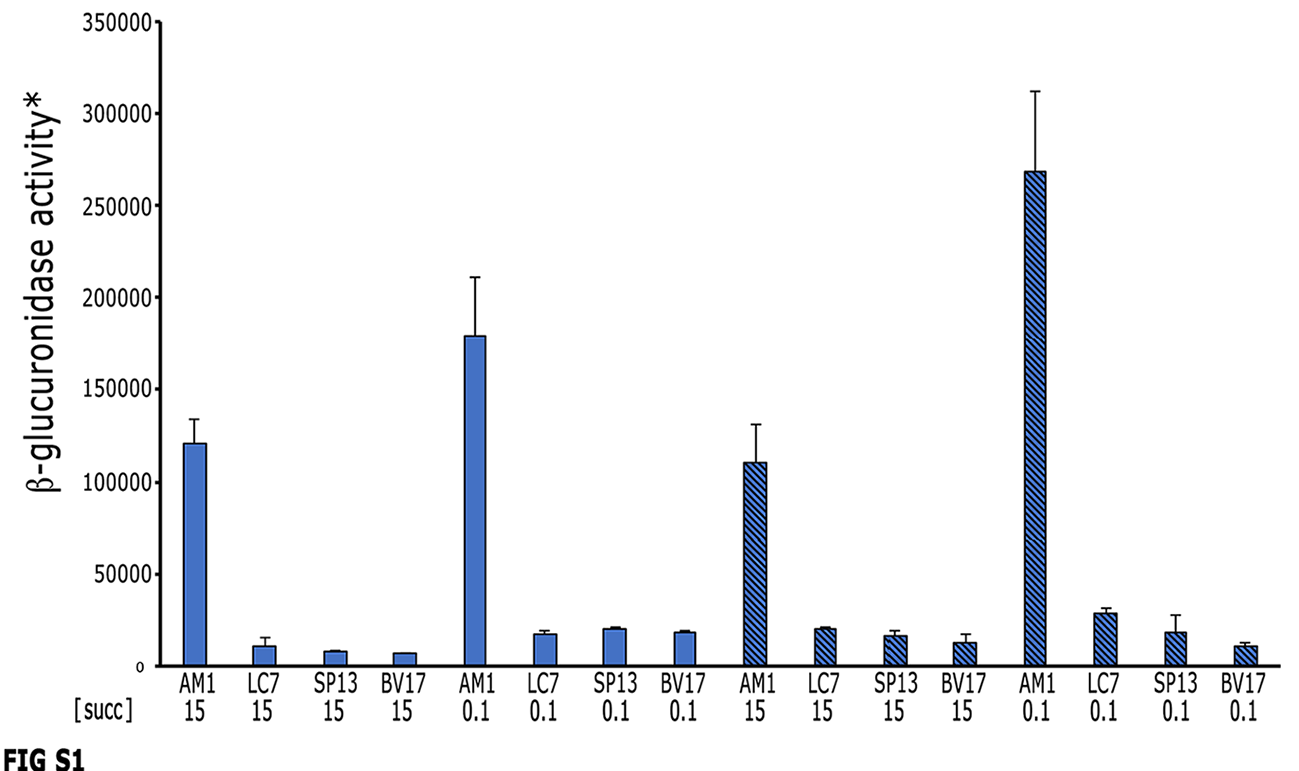

Supplement: FIG S1 [file mbio.01481-22-s0002.tif]

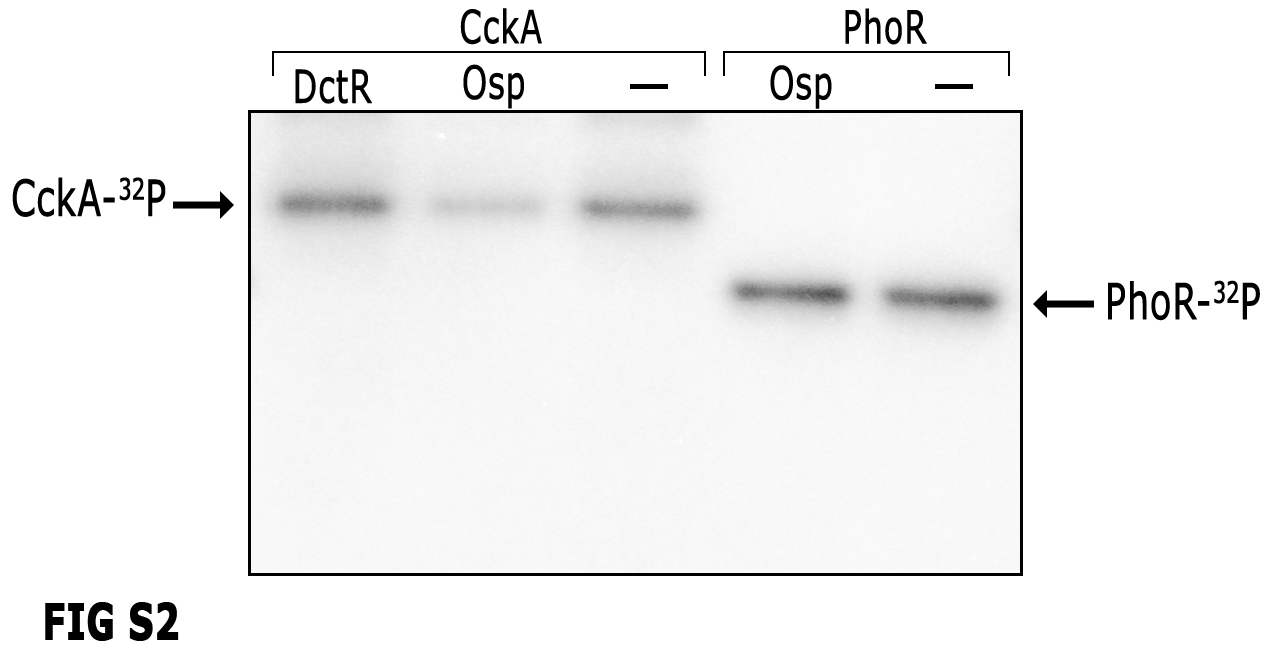

Supplement: FIG S2 [file mbio.01481-22-s0003.tif]

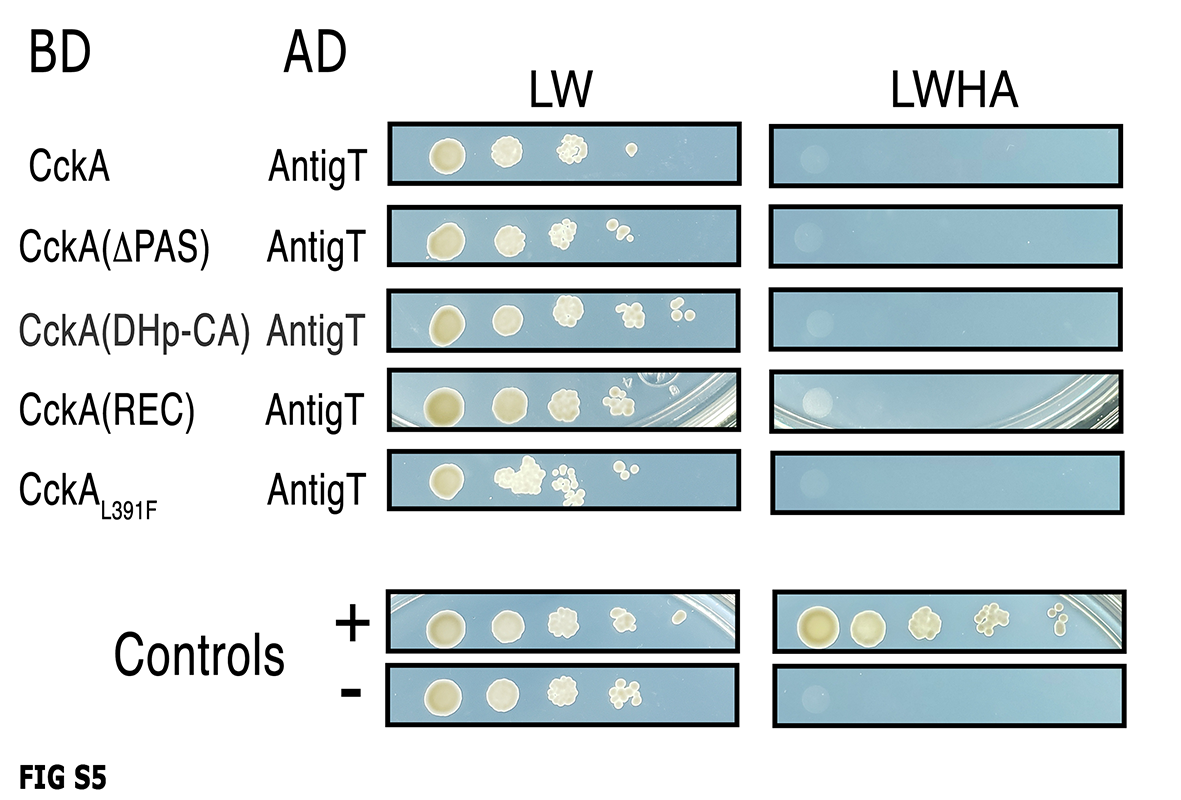

Supplement: FIG S5 [file mbio.01481-22-s0006.tif]

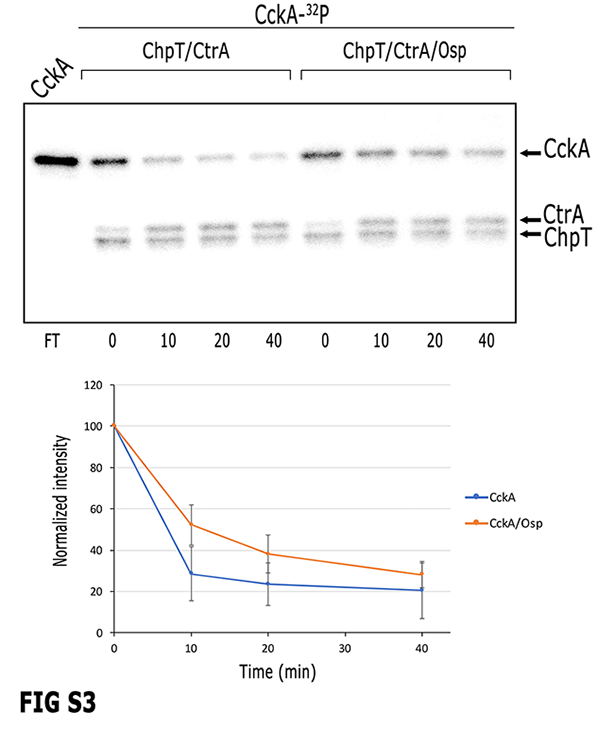

Supplement: FIG S3 [file mbio.01481-22-s0004.tif]

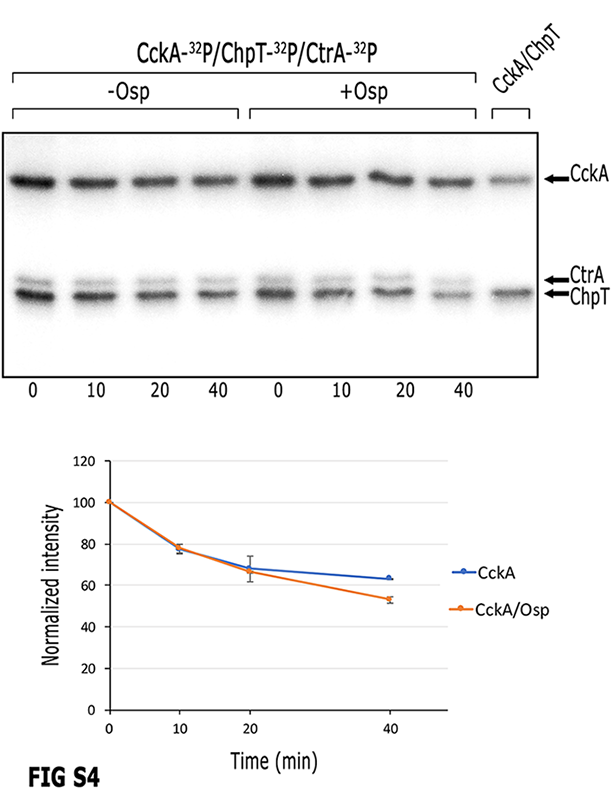

Supplement: FIG S4 [file mbio.01481-22-s0005.tif]

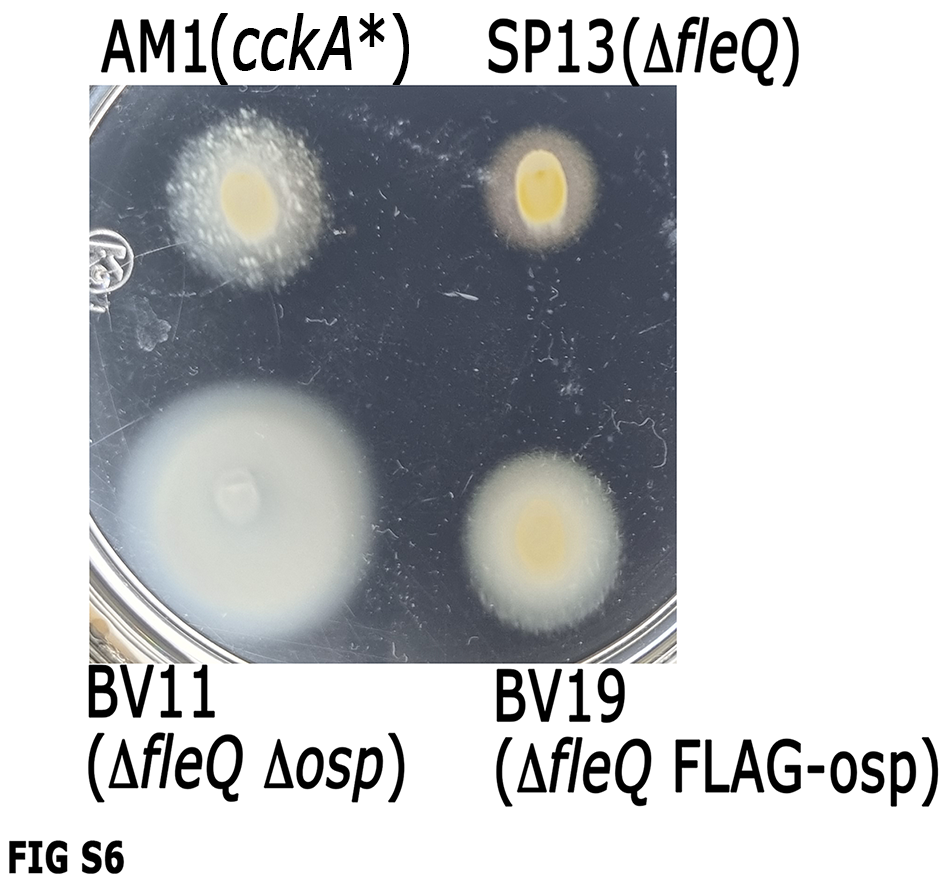

Supplement: FIG S6 [file mbio.01481-22-s0007.tif]
