# Supplementary material for: The Histidine Kinase CckA Is Directly Inhibited by a Response Regulator-like Protein in a Negative Feedback Loop
Source: mBio. 2022 Jul 25;13(4):e01481-22. doi: 10.1128/mbio.01481-22 (PMC9430884; doi:10.1128/mbio.01481-22)
Supplement: TABLE S2 [file mbio.01481-22-s0010.docx]

Table S2 Species and accession numbers of the sequences used to obtain the phylogenetic tree

| Strain | Tree name | Genome accesion number | RpoC | Osp | DivK |
| --- | --- | --- | --- | --- | --- |
| *Actibacterium atlanticum 22II-S11-z10* | Actibacterium_atlanticum | GCA_000671395.1 | KCV80777 |  |  |
| *Actibacterium pelagium JN33* | Actibacterium_pelagium | GCA_002285415.1 | WP_095593921 |  |  |
| *Aestuariivita boseongensis BS-B2* | Aestuariivita_boseongensis | GCA_001262635.1 | WP_050931493 | WP_050928652 |  |
| Albidovulum inexpectatum DSM 12048 | Albidovulum_inexpectatum | GCA_002927635.1 | PPB79601 |  |  |
| *Albimonas pacifica CGMCC 1.11030* | Albimonas_pacifica | GCA_900113695.1 | SFI00853 |  |  |
| *Amylibacter kogurei strain 4G11* | Amylibacter_kogurei | GCA_002742285.1 | PIB25196 | PIB23191 |  |
| *Brevundimonas diminuta ATCC(B) 19146* | Brevundimonas_diminuta | GCA_004102925.1 | WP_076224664 |  | WP_003164449 |
| *Brevundimonas vesicularis FDAARGOS_289* | Brevundimonas_vesicularis | GCA_002208825.2 | WP_055754901 |  | ASE40561 |
| *Caulobacter vibrioides NA 1000* | Caulobacter_vibrioides | GCA_000022005.1 | YP_002515910 |  | YP_002517920 |
| *Celeribacter baekdonensis DSM 27375* | Celeribacter_baekdonensis | GCA_900102315.1 | WP_066707218 | SDF70266 |  |
| *Celeribacter ethanolicus NH195* | Celeribacter_ethanolicus | GCA_001550095.1 | SFK27297 | WP_066707909 |  |
| *Celeribacter manganoxidans DY25* | Pacificitalea_manganoxidans | GCA_002504165.1 | ATI42951 | ATI43176 |  |
| *Celeribacter neptunius DSM 26471* | Celeribacter_neptunius | GCA_900113955.1 | PTR18330 | SFK13407 |  |
| *Cereibacter changlensis DSM 18774* | Cereibacter_changlensis | GCA_003254335.1 | ODM41211 | PZX58697 |  |
| *Cognatiyoonia koreensis DSM 17925* | Cognatiyoonia_koreensis | GCA_900109295.1 | SEW47652 | SEW38493 |  |
| *Cognatiyoonia sediminum DSM 28715* | Cognatiyoonia_sediminum | GCA_900129845.1 | SHH27645 | SHH04478 |  |
| *Defluviimonas alba cai42* | Frigidibacter_mobilis | GCA_001620265.1 | AMY67679 | SMY06504 |  |
| *Defluviimonas aquaemixtae CECT 8626* | Defluviimonas_aquaemixtae | GCA_900302475.1 | SPH25099 | SPH16573 |  |
| *Defluviimonas denitrificans DSM 18921* | Defluviimonas_denitrificans | GCA_002973535.1 | PQV55337 | PQV55803 |  |
| *Dinoroseobacter shibae DFL 12* | Dinoroseobacter_shibae | GCA_000018145.1 | ABV92018 | ABV91917 |  |
| *Epibacterium mobile DSM 23403* | Epibacterium_mobile | GCA_900106635.1 | WP_009176501 |  |  |
| *Flavimaricola marinus CECT 8899* | Flavimaricola_marinus | GCA_900184895.1 | SMY06435 |  |  |
| *Gemmobacter aquatilis DSM 3857* | Gemmobacter_aquatilis | GCA_900110025.1 | SEO31615 |  |  |
| *Gemmobacter megaterium DSM 26375* | Wagnerdoeblera_megaterium | GCA_900156815.1 | WP_028031864 |  |  |
| *Gemmobacter nectariphilus DSM 15620* | Wagnerdoeblera_nectariphilus | GCA_000429765.1 | SIS52537 |  |  |
| *Haematobacter massiliensis CCUG 47968* | Haematobacter_massiliensis | GCA_000740795.1 | KFI25179 |  |  |
| *Haematobacter missouriensis H1892* | Haematobacter_missouriensis | GCA_002196895.1 | OWJ74397 |  |  |
| *Henriciella marina DSM 19595* | Henriciella_marina | GCA_000376805.1 | WP_018146781 |  | WP_040500691 |
| *Hyphomonas atlantica 22II1-22F38* | Hyphomonas_atlantica | GCA_000682715.1 | KCZ61886 |  | KCZ60428 |
| *Hyphomonas neptunium ATCC15444* | Hyphomonas_neptunium | GCA_000013025.1 | ABI76613 |  | ABI78279 |
| *Jannaschia aquimarina GSW-M26* | Jannaschia_aquimarina | GCA_000877395.1 | KIT15263 | KIT16437 |  |
| *Jannaschia helgolandensis DSM 14858* | Jannaschia_helgolandensis | GCA_900109285.1 | SEL80859 | SEK67075 |  |
| *Jhaorihella thermophila DSM 23413* | Jhaorihella_thermophila | GCA_900108275.1 | SEG11334 | SEF81904 |  |
| *Leisingera aquimarina DSM 24565* | Leisingera_aquimarina | GCA_000473165.1 | WP_027257334 |  |  |
| *Leisingera caerulea DSM 24564* | Leisingera_caerulea | GCA_000473325.1 | WP_027236234 |  |  |
| *Leisingera daeponensis DSM 23529* | Leisingera_daeponensis | GCA_000473145.1 | WP_008557437 |  |  |
| *Leisingera methylohalidivorans DSM 14336* | Leisingera_methylohalidivorans | GCA_000511355.1 | AHC99464 |  |  |
| *Limimaricola cinnabarinus LL-001* | Limimaricola_cinnabarinus | GCA_000466965.1 | GAD57028 |  |  |
| *Limimaricola hongkongensis DSM 17492* | Limimaricola_hongkongensis | GCA_000600975.2 | EYD73484 |  |  |
| *Litoreibacter albidus DSM 26922* | Litoreibacter_albidus | GCA_900107015.1 | SDX68687 | SDW66021 |  |
| *Litoreibacter arenae DSM 19593* | Litoreibacter_arenae | GCA_000442275.2 | EPX76904 | EPX77261 |  |
| *Litoreibacter janthinus DSM 26921* | Litoreibacter_janthinus | GCA_900111945.1 | SFR65490 | SFR33360 |  |
| *Litoreibacter meonggei DSM 29466* | Litoreibacter_meonggei | GCA_003663885.1 | RLJ40761 | RLJ41125 |  |
| *Litorimicrobium taeanense DSM 22007* | Litorimicrobium_taeanense | GCA_900110775.1 | SEQ04336 | SEP84051 |  |
| *Loktanella atrilutea DSM 29326* | Loktanella_atrilutea | GCA_900128995.1 | SHF20753 | SHE82228 |  |
| *Loktanella fryxellensis DSM 16213* | Loktanella_fryxellensis | GCA_900110065.1 | SEN76788 | SEM75348 |  |
| *Loktanella salsilacus DSM 16199* | Loktanella_salsilacus | GCA_900114485.1 | SFL66905 | SFL47365 |  |
| *Maricaulis maris MCS10* | Maricaulis_maris | GCA_000014745.1 | ABI66093 |  | ABI65568 |
| *Marinovum algicola FF3* | Marinovum_algicola | GCA_900109145.1 | WP_074840290 | SEJ56271 |  |
| *Maritimibacter alkaliphilus HTCC2654* | Maritimibacter_alkaliphilus | GCA_008124775.1 | EAQ13073 | EAQ13089 |  |
| *Maritimibacter harenae DP07* | Maritimibacter_harenae | GCA_009882975.1 | MZR15458 | MZR13594 |  |
| *Marivita cryptomonadis CL-SK44* | Marivita_cryptomonadis | GCA_002115725.1 | WP_085628840 | WP_085627934 |  |
| *Marivita geojedonensis DPG-138* | Marivita_geojedonensis | GCA_002115805.1 | OSQ44098 | OSQ53142 |  |
| *Marivita hallyeonensis DSM 29431* | Marivita_hallyeonensis | GCA_900129875.1 | SHI07924 | SHH97511 |  |
| *Monaibacterium marinum C7* | Monaibacterium_marinum | GCA_900231835.1 | SOH94638 |  |  |
| *Neptunicoccus sediminis CY02* | Neptunicoccus_sediminis | GCA_001719615.1 | WP_083225714 | WP_069298779 |  |
| *Nereida ignava CECT 5292* | Nereida_ignava | GCA_001049735.1 | CRK76475 | CRK74950 |  |
| *Nioella nitratireducens SSW136* | Nioella_nitratireducens | GCA_001879715.1 | WP_071673921 |  |  |
| *Oceanicella actignis CGMCC 1.10808* | Oceanicella_actignis | GCA_900143155.1 | SHN73248 |  |  |
| *Oceanicola granulosus HTCC2516* | Oceanicola_granulosus | GCA_000153305.1 | EAR49402 |  |  |
| *Oceanicola litoreus DSM 29440* | Vannielia_litorea | GCA_900142295.1 | SIO22111 | SIO31853 |  |
| *Oceaniovalibus guishaninsula JLT2003* | Oceaniovalibus_guishaninsula | GCA_000299575.1 | EKE45246 |  |  |
| *Octadecabacter antarcticus 307* | Octadecabacter_antarcticus | GCA_000155675.2 | AGI69804 |  |  |
| *Octadecabacter arcticus 238* | Octadecabacter_arcticus | GCA_000155735.2 | AGI70526 |  |  |
| *Paenirhodobacter enshiensis DW2-9* | Sinirhodobacter_enshiensis | GCA_000740785.1 | KFI25542 |  |  |
| *Paracoccus aminophilus JCM 7686* | Paracoccus_aminophilus | GCA_000444995.1 | AGT10255 |  |  |
| *Paracoccus pantotrophus DSM 2944* | Paracoccus_pantotrophus | GCA_008824185.1 | QFG37979 |  |  |
| *Paracoccus zeaxanthinifaciens ATCC 21588* | Paracoccus_zeaxanthinifaciens | GCA_000420145.1 | WP_022705690 |  |  |
| *Pararhodobacter aggregans D1-19* | Pararhodobacter_aggregans | GCA_003075525.1 | PVE46209 |  |  |
| *Pararhodobacter marinus CIC4N-9* | Pararhodobacter_marinus | GCA_003122215.1 | PWE27682 |  |  |
| *Phaeobacter gallaeciensis DSM 26640* | Phaeobacter_gallaeciensis | GCA_000511385.1 | AHD08079 |  |  |
| *Phaeobacter inhibens DSM 16374* | Phaeobacter_inhibens | GCA_000473105.1 | WP_014876024 |  |  |
| *Planktomarina temperata RCA23* | Planktomarina_temperata | GCA_000738435.1 | AII88242 |  |  |
| *Pontivivens insulae CECT 8812* | Pontivivens_insulae | GCA_900302495.1 | SPF27778 |  |  |
| *Pseudodonghicola xiamenensis DSM 18339* | Donghicola_xiamenensis | GCA_000429365.1 | WP_028094067 | WP_028092610 |  |
| *Pseudooceanicola batsensis HTCC2597* | Pseudooceanicola_batsensis | GCA_000152725.1 | EAQ04072 | EAQ04522 |  |
| *Pseudophaeobacter arcticus DSM 23566* | Pseudophaeobacter_arcticus | GCA_000473205.1 | WP_027238987 |  |  |
| *Pseudorhodobacter antarcticus CGMCC 1.10836* | Pseudorhodobacter_antarcticus | GCA_900110135.1 | SEN21862 |  |  |
| *Pseudorhodobacter aquimaris KCTC 23043* | Pseudorhodobacter_aquimaris | GCA_001202025.1 | WP_050526161 |  |  |
| *Pseudorhodobacter ferrugineus DSM 5888* | Pseudorhodobacter_ferrugineus | GCA_000420745.1 | WP_022705587 |  |  |
| *Pseudoruegeria haliotis DSM 29328* | Aliiruegeria_haliotis | GCA_003003255.1 | PRY21164 | PRY21154 |  |
| *Pseudoruegeria lutimaris DSM 25294* | Aliiruegeria_lutimaris | GCA_900099935.1 | SDL19643 | SDI72855 |  |
| *Pseudoruegeria marinistellae SF-16* | Pseudoruegeria_marinistellae | GCA_001509585.1 | WP_068117056 | WP_068118168 |  |
| *Puniceibacterium sediminis DSM 29052* | Puniceibacterium_sediminis | GCA_900188035.1 | SNR33780 | SNR78869 |  |
| *Rhodobaca barguzinensis alga05* | R_bogoriensis_barguzinensis | GCA_001870665.2 | ATX65164 |  |  |
| *Rhodobacter aestuarii DSM 19945* | Rhodobacter_aestuarii | GCA_900156655.1 | SIT14655 |  |  |
| *Rhodobacter azotoformans KA25* | Cereibacter_azotoformans | GCA_003050905.1 | WP_107664828 | PTR20745 |  |
| *Rhodobacter blasticus DSM 2131* | Fuscovulum_blasticum | GCA_003034965.1 | PTE12759 | AMY67619 |  |
| *Rhodobacter capsulatus DSM 1710* | Rhodobacter_capsulatus | GCA_003254295.1 | WP_055209135 |  |  |
| *Rhodobacter johrii JA192* | Cereibacter_johrii | GCA_003046325.1 | SNX68436 | ODM43394 |  |
| *Rhodobacter maris JA276* | Rhodobacter_maris | GCA_900217815.1 | SOB94876 |  |  |
| *Rhodobacter megalophilus DSM 18937* | C_sphaeroides_megalophilum | GCA_900188265.1 | SDG50138 | SNS57577 |  |
| *Rhodobacter ovatus JA234* | Cereibacter_ovatus | GCA_900207575.1 | ABA77855 | SNX72866 |  |
| *Rhodobacter sphaeroides 2.4.1* | Cereibacter_sphaeroides_2.4.1 | GCA_003324715.1 | SNT06771 | ABA80052 |  |
| *Rhodobacter sphaeroides WS8N* | Cereibacter_sphaeroides_WS8N | GCA_000212605.1 | *EGJ23160* | EGJ22365 |  |
| *Rhodobacter veldkampii DSM 11550* | Phaeovulum_veldkampii | GCA_004363195.1 | MBK5946872 |  |  |
| *Rhodobacter vinaykumarii JA123* | Phaeovulum_vinaykumarii | GCA_900217755.1 | SOC01150 |  |  |
| *Rhodobacter viridis JA737* | Rhodobacter_viridis | GCA_003217355.1 | PYF10957 |  |  |
| *Rhodovulum imhoffii DSM 18064* | Rhodovulum_imhoffii | GCA_003046545.1 | MBK5934866 |  |  |
| *Rhodovulum robiginosum DSM 12329* | Rhodovulum_robiginosum | GCA_003944755.1 | RSK40797 |  |  |
| *Rhodovulum sulfidophilum DSM 1374* | Rhodovulum_sulfidophilum | GCA_001633165.1 | ANB32827 |  |  |
| *Roseibaca calidilacus HL-91* | Roseibaca_calidilacus | GCA_001517585.1 | CUX79508 |  |  |
| *Roseibaca ekhonensis CECT 7235* | Roseibaca_ekhonensis | GCA_900499075.1 | SUZ33085 |  |  |
| *Roseibacterium elongatum DSM 19469* | Roseibacterium_elongatum | GCA_000590925.1 | AHM03926 |  |  |
| *Roseicitreum antarcticum CGMCC 1.8894* | Roseicitreum_antarcticum | GCA_014681765.1 | SDX78784 |  |  |
| *Roseicyclus mahoneyensis DSM 16097* | Roseicyclus_mahoneyensis | GCA_003148775.1 | PWK59086 |  |  |
| *Roseobacter denitrificans OCh 114 DSM 7001* | Roseobacter_denitrificans | GCA_900113215.1 | ABG33465 | ABG30975 |  |
| *Roseobacter litoralis Och 149* | Roseobacter_litoralis | GCA_000154785.2 | AEI92452 | AEI95393 |  |
| *Roseovarius aestuarii CECT 7745* | Roseovarius_aestuarii | GCA_900172285.1 | SMC13673 | SMC10772 |  |
| *Roseovarius albus CECT 7450* | Roseovarius_albus | GCA_900172335.1 | SLN32545 |  |  |
| *Roseovarius atlanticus R12B* | Roseovarius_atlanticus | GCA_001441615.1 | KRS10392 | KRS14116 |  |
| *Roseovarius marisflavi DSM 29327* | Roseovarius_marisflavi | GCA_900142625.1 | SHL62188 | SHL07066 |  |
| *Roseovarius mucosus DSM 17069* | Roseovarius_mucosus | GCA_000768555.3 | KGM88858 |  |  |
| *Ruegeria atlantica CECT 4292* | Ruegeria_atlantica | GCA_001458195.1 | CUH50561 | CUH49563 |  |
| *Ruegeria conchae TW15* | Ruegeria_conchae | GCA_000192475.2 | RLK07784 | RLK08113 |  |
| *Ruegeria halocynthiae DSM 27839* | Ruegeria_halocynthiae | GCA_900106805.1 | SDX50253 | SDX57655 |  |
| *Ruegeria pomeroyi DSS-3* | Ruegeria_pomeroyi | GCA_000011965.2 | AAV96732 | AAV96458 |  |
| *Ruegeria sp. TM1040* | Epibacterium_TM1040 | GCA_000014065.1 | *ABF62968* |  |  |
| *Sagittula stellata E-37* | Sagittula_stellata | GCA_000169415.1 | EBA06220 | EBA10546 |  |
| *Salinihabitans flavidus DSM 27842* | Salinihabitans_flavidus | GCA_900110425.1 | SEO74159 | SEP07844 |  |
| *Salipiger marinus DSM 26424* | Salipiger_marinus | GCA_900100085.1 | SDJ50631 | SDI13184 |  |
| *Salipiger mucosus DSM 16094* | Salipiger_mucosus | GCA_000442255.1 | EPX86859 | EPX87036 |  |
| *Salipiger thiooxidans DSM 10146* | Salipiger_thiooxidans | GCA_900102075.1 | SDF60425 | SDF14621 |  |
| *Sedimentitalea nanhaiensis DSM 24252* | Sedimentitalea_nanhaiensis | GCA_000473225.1 | SFU18154 | SFT33342 |  |
| *Sediminimonas qiaohouensis DSM 21189* | Sediminimonas_qiaohouensis | GCA_000423645.1 | WP_026759055 | MTJ05468 |  |
| *Shimia abyssi DSM 100673* | Shimia_abyssi | GCA_003014475.1 | PSL14268 | PSL17838 |  |
| *Shimia aestuarii DSM 15283* | Shimia_aestuarii | GCA_900114635.1 | SFM75650 | SFL67127 |  |
| *Sulfitobacter indolifex HEL-45* | Sulfitobacter_indolifex | GCA_000172095.1 | EDQ06754 |  |  |
| *Sulfitobacter pseudonitzschiae H3* | Pseudosulfitobacter_pseudonitzsc | GCA_000712315.1 | KEJ93792 | KEJ95091 |  |
| *Tabrizicola aquatica RCRI19* | Tabrizicola_aquatica | GCA_002900975.1 | WP_103259205 |  |  |
| *Thalassobacter stenotrophicus CECT 5294* | Thalassobacter_stenotrophicus | GCA_001458315.1 | CUH59300 | CUH61837 |  |
| *Thalassobius autumnalis CECT 5118* | Thalassobius_autumnalis | GCA_001458255.1 | CUH64467 |  |  |
| *Thalassobius gelatinovorus CECT 4357* | Thalassobius_gelatinovorus | GCA_001458355.1 | CUH65788 |  |  |
| *Thalassobius mediterraneus CECT 5383* | Thalassobius_mediterraneus | GCA_001458435.1 | CUH83498 |  | QYJ00542 |
| *Thalassococcus halodurans DSM 26915* | Thalassococcus_halodurans | GCA_900108225.1 | SEG52122 | SEF72868 |  |
| *Thioclava atlantica 13D2W-2* | Thioclava_atlantica | GCA_000737065.1 | KFE33472 |  |  |
| *Thioclava electrotropha Elox9* | Thioclava_electrotropha | GCA_002085925.2 | QPZ92658 |  |  |
| *Thioclava pacifica DSM 10166* | Thioclava_pacifica | GCA_000714535.1 | KEO54608 |  |  |
| *Tropicibacter naphthalenivorans DSM 19561* | Tropicibacter_naphthalenivorans | GCA_900176475.1 | SMD10375 | SMC79686 |  |
| *Tropicibacter phthalicicus CECT 8649* | Tropicibacter_phthalicicus | GCA_900184825.1 | SMX29721 | SMX26862 |  |
| *Tropicimonas sediminicola DSM 29339* | Tropicimonas_sediminicola | GCA_900188335.1 | SNT38898 | SNS88829 |  |
| *Wenxinia marina DSM 24838* | Wenxinia_marina | GCA_000836695.1 | KIQ67536 | KIQ68055 |  |
| *Wenxinia saemankumensis DSM 100565* | Wenxinia_saemankumensis | GCA_900141735.1 | SHI56608 | SHI84376 |  |
| *Yoonia litorea DSM 29433* | Yoonia_litorea | GCA_900114675.1 | SFS22108 | SFS21443 |  |
| *Yoonia maricola DSM 29128* | Yoonia_maricola | GCA_002797915.1 | PJI85399 | PJI92990 |  |
| *Yoonia sediminilitoris DSM 29955* | Yoonia_sediminilitoris | GCA_003058085.1 | PUB11492 | PUB14161 |  |
| *Yoonia vestfoldensis DSM 16212* | Yoonia_vestfoldensis | GCA_000382265.1 | WP_019956812 | ART99670 |  |
