# Supplementary material for: The Histidine Kinase CckA Is Directly Inhibited by a Response Regulator-like Protein in a Negative Feedback Loop
Source: mBio. 2022 Jul 25;13(4):e01481-22. doi: 10.1128/mbio.01481-22 (PMC9430884; doi:10.1128/mbio.01481-22)
Supplement: FIG S7 [file mbio.01481-22-s0008.docx]

A

B

------------------------------------------------------------------

Motif TTAACCHTTYGTTAA MEME-1 block diagrams

------------------------------------------------------------------

SEQUENCE NAME POSITION P-VALUE MOTIF DIAGRAM

------------- ---------------- -------------

Pseudosulfitobacter_pseu 9.7e-10 114_[+1]_56

Yoonia_maricola 3.9e-09 102_[+1]_82

Yoonia_litorea 3.9e-09 97_[+1]_77

Ruegeria_halocynthiae 4.9e-09 96_[+1]_69

Ruegeria_conchae 4.9e-09 96_[+1]_70

Ruegeria_atlantica 4.9e-09 98_[+1]_69

Roseovarius_marisflavi 4.9e-09 92_[+1]_71

Roseovarius_aestuarii 4.9e-09 237_[+1]_85

Litorimicrobium_taeanens 6.8e-09 87_[+1]_65

Shimia_abyssi 1.5e-08 87_[+1]_68

Litoreibacter_albidus 1.5e-08 98_[+1]_124

Aliiruegeria_lutimaris 1.5e-08 100_[+1]_59

Roseovarius_atlanticus 2.5e-08 194_[+1]_79

Tropicimonas_sediminicol 3.3e-08 113_[+1]_61

Aliiruegeria_haliotis 3.3e-08 95_[+1]_61

Jhaorihella_thermophila 3.9e-08 98_[+1]_72

Yoonia_sediminilitoris 4.9e-08 102_[+1]_107

Shimia_aestuarii 7.1e-08 76_[+1]_78

Sedimentitalea_nanhaiens 7.1e-08 103_[+1]_71

Amylibacter_kogurei 7.1e-08 75_[+1]_43

Tropicibacter_phthalicic 8.1e-08 115_[+1]_55

Cognatiyoonia_koreensis 8.1e-08 97_[-1]_85

Litoreibacter_janthinus 1e-07 98_[+1]_103

Aestuariivita_boseongens 1e-07 86_[+1]_69

Flavimaricola_marinus 1.1e-07 110_[+1]_78

Defluviimonas_aquaemixta 1.3e-07 85_[+1]_75

Cereibacter_azotoformans 1.3e-07 389_[-1]_54

Litoreibacter_meonggei 1.5e-07 98_[+1]_104

Litoreibacter_arenae 1.5e-07 97_[+1]_104

Pseudoruegeria_mariniste 1.7e-07 134_[+1]_60

Roseobacter_litoralis 1.9e-07 115_[+1]_69

Roseobacter_denitrifican 1.9e-07 115_[+1]_66

Marivita_hallyeonensis 2.1e-07 115_[+1]_58

Marivita_cryptomonadis 2.3e-07 125_[+1]_60

Cereibacter_sphaeroides_ 2.8e-07 175_[-1]_53

Cereibacter_sphaeroides_ 2.8e-07 175_[-1]_53

Cereibacter_johrii 2.8e-07 183_[-1]_53

C_sphaeroides_megalophil 2.8e-07 175_[-1]_53

Ruegeria_pomeroyi 3.1e-07 94_[+1]_76

Neptunicoccus_sediminis 4.3e-07 80_[+1]_43

Marivita_geojedonensis 4.7e-07 130_[+1]_56

Sediminimonas_qiaohouens 5.2e-07 105_[+1]_76

Salipiger_marinus 5.7e-07 132_[+1]_70

Marinovum_algicola 5.7e-07 136_[+1]_77

Cereibacter_changlensis 7.4e-07 101_[-1]_48

Tropicibacter_naphthalen 8.1e-07 118_[+1]_59

Cereibacter_ovatus 8.1e-07 73_[+1]_54

Thalassobacter_stenotrop 8.9e-07 138_[+1]_79

Wenxinia_marina 1.1e-06 92_[+1]_78

Salipiger_thiooxidans 1.1e-06 106_[+1]_63

Jannaschia_aquimarina 1.5e-06 94_[+1]_57

Jannaschia_helgolandensi 1.7e-06 117_[+1]_66

Defluviimonas_denitrific 2.5e-06 86_[+1]_72

Pseudooceanicola_batsens 4.6e-06 50_[+1]_81

Celeribacter_ethanolicus 5.4e-06 92_[+1]_79

Celeribacter_baekdonensi 5.8e-06 113_[-1]_56

Cognatiyoonia_sediminum 6.2e-06 100_[+1]_84

Pacificitalea_manganoxid 1.3e-05 196_[-1]_86

Maritimibacter_alkaliphi 3.4e-05 96_[-1]_75

Frigidibacter_mobilis 3.4e-05 121_[-1]_63

Sagittula_stellata 3.6e-05 107_[+1]_56

Celeribacter_neptunius 3.6e-05 111_[+1]_120

Maritimibacter_harenae 4.3e-05 94_[+1]_69

Nereida_ignava 5.3e-05 80_[-1]_55

------------------------------------------------------------------

**FIG S7.** Analysis of the intergenic upstream regions of the *osp* orthologs using MEME. A) The red boxes represent the best motif found by MEME with the consensus TTAACCHTTYGTTAA and their respective e-values for each sequence. B) MEME results showing at the far-right column, the distance between the motif and the ATG start codon of *osp.*
