# Supplementary material for: The Histidine Kinase CckA Is Directly Inhibited by a Response Regulator-like Protein in a Negative Feedback Loop
Source: mBio. 2022 Jul 25;13(4):e01481-22. doi: 10.1128/mbio.01481-22 (PMC9430884; doi:10.1128/mbio.01481-22)
Supplement: TABLE S1 [file mbio.01481-22-s0009.docx]

Table S1 Oligonucleotides used in this work

| FwUPospEco | GCGAATTCCGCTCGGTCACTTCTGGGAC |
| --- | --- |
| RvUPospBam | GCGGATCCCTCCACGATCAGGACGTGCAT |
| FwDWospBam | GCGGATCCGACGGGTCCATCTTCACCCAT |
| RvDWospXba | GCTCTAGACTCGAGCCAGAAATCGAGCG |
| Fw_hygrouniv | GCGGATCCCGGGCCAGCTCCGCCATCGCC |
| Rv_hygrouniv | GCGGATCCGGCGGCCCGGGGCGTCAGGC |
| FWospQE | GCCCATGGACGTCCTGATCGTGGAGAC |
| Rv osp Hind | GCAAGCTTGGCCGCGTGAACGTAATGCTC |
| Fw Ccka cristal NdeI | GCCATATGAAGACGCTCGAGGCTCAGTTC |
| Rv CckA cristal XbaI | GCTCTAGATCACGGCTCTTCATTGATGGG |
| FWospQE | GCCCATGGACGTCCTGATCGTGGAGAC |
| Rv osp pBAD HindIII | GCAAGCTTCTCAGGCCGCGTGAACGTAATG |
| T7 prom BglII | GCAGATCTTTAATACGACTCACTATAGGG |
| T7 ter BglII | GCAGATCTGATATAGTTCCTCCTTTCAGC |
| Fw CckA DH NdeI | GCCATATGCTCGATCTGCGGTTCGCGC |
| 454 B | GGAGCTCGGCTCAACGTCTCGCCCTGT |
| Fw DPasCckA DH NdeI | GCCATATGGATGTCGCCGCCGAGCGGATG |
| Rv CckADrec NcoI | GCCCATGGTCACTCGACGGGAAGCGGCTC |
| Fw CckA REC NdeI | GCCATATGGAAGAGCCGCTTCCCGTCGAG |
| Fw osp NdeI | GCCATATGCACGTCCTGATCGTG |
| Rv OspDH BamHI | GCGGATCCCTCAGGCCGCGTGAACGTAATG |
| Rv Met osp Eco | GCGAATTCCATTCTGGCTTCCTTCGACTTTG |
| Rv Flag Nde | GCCATATGTTTATCGTCGTCATCTTTGTAG |
| Fw osp Nde | GCCATATGCACGTCCTGATCGTG |
| Fw dw ospflag Bam | GCGGATCCCCGCGCAGCATTCGTAACGCC |
| Rv dw ospflag Xba | GCTCTAGATTCGACTCGCGGATCAACGCG |
| Fw_phoR_BamHI | GCGGATCCCTGAACCGGCGAAAGGCCGTC |
| Rv_PhoR_EcoRI | GCGAATTCTCAGGCGCTTCCCGCTTCCGC |
| DctR_ΔHTH_EcoRI | GCGAATTCTCAGCGCGCGCCGCTGTGGGCCAG |
| DctRA | CCGGAATTCACAGGGTGCGTCCATATCAT |
| Fw_osp_Hind | GCAAGCTTCTTTTCCCCGCCTCTTTCGG |
| Rv_osp_Xba | GCTCTAGAAGACTGGGTGCGGGCGTTAC |
| Fw ospD51N | CAGGTCGTGATGCTGAATCTGATGCTCGACG |
| Rv ospD51N | CGTCGAGCATCAGATTCAGCATCACGACCTG |
