# Supplementary material for: The Histidine Kinase CckA Is Directly Inhibited by a Response Regulator-like Protein in a Negative Feedback Loop
Source: mBio. 2022 Jul 25;13(4):e01481-22. doi: 10.1128/mbio.01481-22 (PMC9430884; doi:10.1128/mbio.01481-22)
Supplement: TEXT S1 [file mbio.01481-22-s0001.docx]

**Supplementary Methods**

**Strains isolated in this work.** To obtain Δ*osp*::Hyg allele, the upstream and downstream regions of osp were amplified by PCR using the oligonucleotides FwUPospEco and RvUPospBam, and FwDWospBam and RvDWospXba (Table S1) and total DNA from *R. sphaeroides* WS8N. The products of 584 and 534 bp were cloned together through a BamHI site present in the oligonucleotides, and the resultant fragment of 1118 pb was cloned into pTZ18R plasmid to obtain pTZ_ospUPDW. This plasmid was digested with BamHI and ligated with the hygromycin (Hyg^R^) cassette previously digested with BamHI, to obtain pTZBam_Δosp::Hyg. The Hyg cassette was obtained by PCR using the oligonucleotides Fw_hygrouniv and Rv_hygrouniv and the plasmid pIJ963 (1). The fragment carrying the allele Δ*osp::*Hyg was obtained by PCR with the oligonucleotides FwUPospEco and RvDWospXba, and cloned in the SmaI site of pJQ200mp18. The resultant plasmid was introduced in R. sphaeroides by conjugation, and double-recombination events were selected. The correctness of mutants was verified by PCR.

For tagging *osp* with 3XFLAG, a 566 bp PCR product carrying the upstream region of *osp* from the -563 (considering the translation start site as +1) to the start codon of *osp*, was obtained using the oligonucleotides FwUPospEco and RvMetospEco. The fusion between the initial codon of *osp* and the FLAG-tag coding sequence was generated by cloning the 566 bp PCR product into pSUB11 plasmid previously digested with EcoRI. The ligation product was used to amplify by PCR the fragment encompassing the upstream region of *osp* and the coding region of FLAG-tag, for this reaction the oligonucleotides FwUPospEco and RvFlagNde were used. The product was cloned in pTZ18R previously digested with SmaI and subsequently ligated through a NdeI restriction site with the coding region of *osp*, this region was obtained by PCR using the oligonucleotides FwospNde and RvOspDHBamHI. The resulting fragment of 1,000 bp was ligated through a BamHI site to the downstream region of *osp* previously obtained by PCR using the oligonucleotides FwdwospflagBam and RvdwospflagXba. The 1.7 kb product of was ligated to pTZ19RBamHI- in the unique SmaI site to obtain pTZospFlag_1.7. A spectinomycin cassette was obtained by PCR and cloned in a BamHI site of pTZospFlag_1.7. The XbaI fragment from the pTZospFlag_1.7 plasmid containing FLAG-*osp* and the spectinomycin cassette located downstream of the stop codon of *osp*, was subcloned in the suicide plasmid pJQ200mp18 and introduced by conjugation to AM1 or SP13 cells to obtain strains BV18 and BV19, respectively. Double-recombination events were selected, and the correctness of mutants was verified by PCR.

**Protein overexpression and purification.** The coding region of *osp* was amplified by PCR using the oligonucleotides FwospQE and RvospHind. The product was cloned in pET28a to generate the fusion between Osp and the His6x-tag encoded in this vector. The resultant plasmid pET28a_osp-His6x was used to transform *E. coli* Rosetta cells which enable the expression of the Osp-His6x fusion from the T7 promoter. An exponentially growing culture of the transformed strain was induced with 1 mM IPTG for 4 h at 37°C. The cells were collected by centrifugation and suspended in 1/10 of the volume of TEG buffer (50 mM Tris pH 8, NaCl 50 mM and 5% glycerol). The cell suspension was sonicated on ice using a microtip (3 mm), with three bursts of 10 s; and debris was removed by low-speed centrifugation at 5,000 rpm for 2 min. Even though most of the protein was insoluble, a small amount of protein remains soluble; therefore, the supernatant was incubated with Ni-NTA agarose for 2 h at 4°C. The beads were washed three times with PBS pH 7.4. The protein was eluted using PBS/10% glycerol/200 mM imidazole. The cytoplasmic domain of CckA fused to the His6x-tag (His6x-CckA) was purified as described previously using the plasmid pBAD_His-cckA and pBAD_His-cckA F391L (2). The protein His6x-CtrA was obtained using the plasmid pBAD_ctrA and following the procedure previously described (3). His6x-ChpT was obtained using the pBAD_His-chpT plasmid. *E. coli* LMG194 cells transformed with pBAD_His-chpT were grown to mid-exponential phase and the expression of His6x-ChpT was induced with 0.2% arabinose for 4 h at 37°C. The cells were pelleted at 12,000 rpm for 10 min. Cell pellets were suspended in 1/10 of the volume of TEG buffer (50 mM Tris pH 8, NaCl 50 mM and 5% glycerol), sonicated, and centrifuged at 12,000 rpm for 10 min at 4°C to remove cell debris. The supernatant was incubated with Ni-NTA agarose and for 2 h at 4°C. Agarose beads were washed three times with PBS pH 7.4. The protein was eluted with PBS/10% glycerol/200 mM imidazole. Before the phosphorylation assays the purified protein was dialyzed against PBS-10% glycerol buffer for 2 h. To obtain His6X-PhoR, the DNA region encoding the cytoplasmic domain of PhoR was amplified by PCR using the oligonucleotides Fw_phoR BamHI and Rv_phoR_EcoRI. The 1248 bp product was purified and cloned into pET28a. The resultant plasmid was introduced to *E. coli* Rosetta cells and an exponentially growing culture was induced with 1 mM IPTG for 2 h at 37°C. The protein was purified using Ni-NTA agarose and the soluble fraction obtained from total cell extracts obtained by sonication in PBS buffer pH 7.4 at 4°C. We used the previously published protocol to obtain the protein His6x-DctR (4).

**Protein copurification.** To obtain pET28a_His6x-cckA the PCR product carrying the CckA transmitter domain was amplified with Fw CckA cristal NdeI and Rv CckA cristal XbaI and cloned into pTZ18R SmaI, the resultant plasmid was digested with NdeI and BamHI and the insert was subcloned into pET28a previously digested with NdeI and BamHI. To obtain pET28a_osp plasmid, the PCR product carrying *osp* was amplified using the oligonucleotides FWospQE and Rv osp pBAD HindIII and cloned in the pET28a previously digested with NcoI and HindIII. The vector pET28a_His6x-cckA_osp was constructed by cloning the PCR product encompassing *osp* and the upstream and downstream regions that include the T7 promoter and terminator into pET28a_His6x-CckA. For this, an amplification product was obtained using pET28a_Osp and the oligonucleotides T7 prom BglII and T7 ter BglII, this product was cloned into the BglII site of pET28a_6xHis-cckA. Overexpression of His6x-CckA, Osp and His6x-CckA and Osp was done by inducing *E. coli* Rosetta cell cultures carrying pET28a_6xHis-CckA, pET28a_osp or pET28a_His6x-cckA_osp with 1 mM IPTG. His6x-CckA, Osp and His6x-CckA/Osp proteins were purified as described in the previous section. The purified proteins were subjected to SDS-PAGE (5) and stained with Coomassie brilliant blue R-250.

**Plasmid constructions.** Plasmids pGBKT7_cckAΔTM, pGBKT7_cckAΔPas, pGBKT7_cckA DHp-CA, and pGBKT7_cckA Rec were constructed by cloning into plasmid pGBKT7 the amplification products obtained with the oligonucleotides indicated in Table S1. Plasmid pGADT7_osp was constructed by cloning into pGADT7 the coding region of *osp* obtained by PCR using the oligonucleotides FwospNdeI and RvospDHBam. pGADT7_REC-dctR plasmid was obtained by cloning the DNA region encoding the REC domain of DctR into pGADT7; for this, the oligonucleotides DctR_ΔHTH_EcoRI and DctRA were used in a PCR reaction. The sequence of these plasmids and the correct fusion was confirmed by sequencing.

pRK_09785 plasmid was obtained by cloning into pRK415 the amplification product of 542 bp generated using the oligonucleotides Fw_osp_HindIII, Rv_osp_Xba and total DNA from WS8N. pRK_09785 D51N (pRK_osp D51N) plasmid was obtained by site-directed mutagenesis using the oligonucleotides Fw ospD51N and Rv ospD51N, following the protocol reported previously (6). To obtain pRK_osp::uidA-aadA, the *uidA*-*aadA* cassette obtained from pWM5 plasmid as a BamHI fragment of 4.2 kb, was cloned into pTZ18R_ospUPDW previously digested with BamHI. The plasmid carrying the reporter gene *uidA* contiguous to the control region of *osp* was selected and digested with XbaI and EcoRI; the 4.7 kb fragment was subcloned into pRK415. The sequence of these plasmids was confirmed by sequencing.

REFERENCES

1. Lydiate DR, Ashby AM, Henderson DJ, Kieser T, Hopwood DA. 1989. Physical and Genetic Characterization of Chromosomal Copies of the *Streptomyces* *coelicolor* Mini-circle. J Gen Microbiol 135:941-955.

2. Vega-Baray B, Domenzain C, Rivera A, Alfaro-Lopez R, Gomez-Cesar E, Poggio S, Dreyfus G, Camarena L. 2015. The flagellar set Fla2 in *Rhodobacter sphaeroides* is controlled by the CckA pathway and is repressed by organic acids and the expression of Fla1. J Bacteriol 197:833-47.

3. Rivera-Osorio A, Osorio A, Poggio S, Dreyfus G, Camarena L. 2018. Architecture of divergent flagellar promoters controlled by CtrA in *Rhodobacter sphaeroides*. BMC Microbiol 18:129.

4. Sanchez-Ortiz VJ, Domenzain C, Poggio S, Dreyfus G, Camarena L. 2021. The periplasmic component of the DctPQM TRAP-transporter is part of the DctS/DctR sensory pathway in *Rhodobacter sphaeroides*. Microbiology (Reading) 167.

5. Laemmli UK. 1970. Cleavage of structural proteins during the assembly of the head of bacteriophage T*4*. Nature 227:680-5.

6. Ho SN, Hunt HD, Horton RM, Pullen JK, Pease LR. 1989. Site-Directed Mutagenesis by Overlap Extension Using the Polymerase Chain-Reaction. Gene 77:51-59.
